# Impacts of GlobalConsent, a Web-Based Social Norms Edutainment Program, on Sexually Violent Behavior and Bystander Behavior Among University Men in Vietnam: Randomized Controlled Trial

**DOI:** 10.2196/35116

**Published:** 2023-01-27

**Authors:** Kathryn M Yount, Yuk Fai Cheong, Irina Bergenfeld, Quach Thu Trang, Jessica M Sales, Yiman Li, Tran Hung Minh

**Affiliations:** 1 Rollins School of Public Health Emory University Atlanta, GA United States; 2 Center for Creative Initiatives in Health and Population Hanoi Vietnam

**Keywords:** behavior change communication, bystander behavior, campus sexual assault, educational entertainment (edutainment), sexual violence, social cognitive theory, social norms theory, Vietnam, mobile phone

## Abstract

**Background:**

*Sexual violence* against women is prevalent worldwide. Prevention programs that treat men as allies and integrate a bystander framework are emerging in lower income settings, but evidence of their effectiveness is conflicting.

**Objective:**

This study aimed to test the impact of GlobalConsent on sexually violent behavior and prosocial bystander behavior among university men in Vietnam.

**Methods:**

We used a double-blind, parallel intervention versus control group design with 1:1 randomization at 2 universities. A total of 793 consenting heterosexual or bisexual men aged 18-24 years who matriculated in September 2019 were enrolled and assigned randomly to GlobalConsent or an attention-control adolescent health education (AHEAD) program. GlobalConsent is an adapted, theory-based, 6-module web-based intervention with diverse behavior change techniques and a locally produced serial drama. AHEAD is a customized, 6-module attention-control program on adolescent health. Both the programs were delivered to computers and smartphones over 12 weeks. Self-reported sexually violent behaviors toward women in the prior 6 months and prosocial bystander behaviors in the prior year were measured at 0, 6, and 12 months.

**Results:**

More than 92.7% (735/793) of men in both study arms completed at least 1 program module, and >90.2% (715/793) of men completed all 6 modules. At baseline, a notable percentage of men reported any sexually violent behavior (GlobalConsent: 123/396, 31.1%; AHEAD: 103/397, 25.9%) in the prior 6 months. Among men receiving GlobalConsent, the odds of reporting a high level (at least 2 acts) of sexually violent behavior at the endline were 1.3 times the odds at baseline. Among men receiving AHEAD, the corresponding odds ratio was higher at 2.7. The odds of reporting any bystander behavior at endline were 0.7 times the odds at baseline for GlobalConsent, and the corresponding odds ratio for AHEAD was lower at 0.5.

**Conclusions:**

Compared with a health attention-control condition, GlobalConsent has sustained favorable impacts on sexually violent behavior and prosocial bystander behavior among matriculating university men in Vietnam, who would otherwise face increasing risks of sexually violent behavior. GlobalConsent shows promise for national scale-up and regional adaptations.

**Trial Registration:**

ClinicalTrials.gov NCT04147455; https://clinicaltrials.gov/ct2/show/NCT04147455

**International Registered Report Identifier (IRRID):**

RR2-10.1186/s12889-020-09454-2

## Introduction

### Background

*Sexual violence* is defined as a sexual act committed against a person when consent is not freely given. Sexual violence ranges from unwanted noncontact sexual experiences to completed forced penetration [1]. Among sexually experienced women aged 15-19 years, forced sexual debut is common worldwide (15%), including in the Asia and Pacific (14%) [2], and women comprise 91% of the victims [3]. The physical, psychological, and economic aftermath of sexual violence often is severe for victims [4,5] and is costly for societies [6].

In Vietnam, sexual violence persists [7,8] despite the legal reforms to address it. Men may discount, excuse, or deny acts of sexual violence [9], and their reported rates of sexually violent behavior (0.2%) are lower than women’s reported rates of victimization (12%) [10]. Thus, prevention with men is crucial to create environments where women’s freedom from violence is possible [11]. However, young men are often difficult to reach [12] and may resist participation in prevention programs [13]. The behavior of bystanders, or witnesses of sexual violence, may also be gender specific [14]. Thus, prevention with men that integrates a bystander framework and treats men as “allies” [15] may address attitudes and behaviors while decreasing resistance to participation [16,17].

However, evidence of programmatic impacts from rigorous evaluations is limited [18]. In an Ovid database search for “sexual violence,” “intervention,” and “review,” we identified 4 reviews published since 2016 that focused on interventions to prevent sexual violence in adolescents and young adults [19-22]. A 2017 review of reviews identified a few interventions focused on boys [19]. A subsequent review of 44 bystander intervention programs in North America found that, on average, programs involved a single session (75%) of <2 hours (mean 116 minutes; range 10-480 minutes) in college populations (75%) as well as in-person presentations (68%) and discussions (54%) with mixed-gender groups (56%). Relatively few interventions were tailored to men (27%) and involved web-based delivery (11%; [20]). Moreover, study designs had important limitations: the majority were not randomized controlled trials (RCTs; 62%) and had small sample sizes (mean 536, range 1-4311) in White populations (73%), high attrition (36%) [20], and short follow-up periods of ≤6 months (89%) focused on nonbehavioral outcomes (66%). A third systematic review focused on interventions to prevent intimate partner, dating, and sexual violence in men and boys and found heterogeneity across the 9 included studies in program content and delivery strategies, study designs, sample sizes, and outcome measurement [21]. Most studies used cluster-randomized designs, recruited undergraduate college students, and evaluated a multisession program delivered via group sessions; however, only 1 program reduced men’s self-reported sexually violent behavior, and most studies were based in the United States. A fourth review that focused on intervention studies to change “hegemonic masculinities” found that 8 of the 10 included quantitative studies were conducted in the United States or Africa, only 1 was web-based, and impacts on sexually violent behavior were mixed [22]. Thus, especially in lower- and middle-income countries (LMICs), gender-specific interventions with men to prevent sexually violent behavior are rare, and theoretically grounded, web-based sexual violence prevention interventions engaging men and involving a bystander framework have not been evaluated in such settings.

This study tested the impact of GlobalConsent on sexually violent behavior and prosocial bystander behavior in university men in Vietnam. The team adapted GlobalConsent from RealConsent, an evidence-based web-delivered intervention tested among university men in the United States [23]. RealConsent is based on formative research [24], social cognitive theory [25], social norms theory [26], and the bystander education model [27]. Sexually violent behavior is theorized to arise from the interplay of sociocontextual factors, personal factors, and behavior. To operationalize this theory of change, programmatic features of RealConsent include gender-specific content [28-31] that resonates with the viewer [32] and diverse behavior change techniques [33], such as providing information and instruction on obtaining effective consent for sex and intervening safely, modeling communication and intervening behaviors, showing positive outcomes for obtaining consent and intervening plus negative outcomes for perpetrating and not intervening, and reinforcing with positive feedback [34]. RealConsent uses didactic presentation of material via integrated audio, video, and infographics; problem-based learning with interactivity and reinforced practice; short videos or animations to model behavior; and educational entertainment, starting and ending each module with a brief episode of a serial drama [23,35].

RealConsent aimed to change 2 primary behaviors by changing 7 cognitive, attitudinal, and affective mediators. The 2 primary behaviors were prosocial intervening behaviors, such as trying to stop a peer from being sexually coercive and sexually violent behavior toward women. The 7 mediators included knowledge of the elements of sexual consent, knowledge and skills to intervene safely, misperceptions of norms about sexual violence and rape, negative attitudes about date rape, positive masculinity, skills in sexual communication, and empathy for the victims of sexual violence. Results from a randomized controlled trial supported the efficacy of RealConsent. A random probability sample of 743 undergraduate men aged 18-24 years attending a large, public, urban university in the Southeastern United States was randomized to RealConsent (376/743, 51%) or a web-based health education attention-control program (367/743, 49%) [23]. Participants were surveyed on the web at baseline, after intervention, and 6 months after intervention. Six 30-minute modules were delivered via a password-protected web portal. At 6 months after intervention, compared with the control group, the RealConsent group intervened more often and engaged in less sexual violence. They reported greater knowledge about legal definitions of sexual assault and elements of effective consent; lower adherence to rape myths, negative date-rape attitudes, hypergender ideology, and hostility to women; greater empathy for rape victims; greater intentions to intervene; less positive outcome expectancies for nonconsensual sex; more positive outcome expectancies for intervening; and less comfort with other men’s inappropriate behavior. The trial experienced high loss to follow-up in the intervention (67%) and control (75%) arms; therefore, results in the retained sample should be interpreted with caution.

### Objectives

To adapt RealConsent for delivery to a new context, we followed the Centers for Disease Control and Prevention’s 5-step process [36]. Step 1, *assess*, involved assessing the target population, the evidence-based intervention (EBI) being considered for implementation, and the implementing agency’s capacity to use the intervention. Step 2, *select*, entailed determining whether to adopt the EBI without adaptations, with adaptations, or to choose another EBI for adaptation. Step 3, *prepare*, involved making the necessary adaptations to the EBI while retaining the core elements. Step 4, *pilot*, entailed piloting the adapted intervention and developing a plan for implementation. We conducted qualitative research to implement steps 1 to 4 [37], and those findings are presented elsewhere [38-40].

To complete step 5, *implement*, we undertook an RCT to test, among men attending 2 universities in Vietnam, the impact of GlobalConsent versus a customized adolescent health education (AHEAD) attention-control condition on changes in 2 primary behavioral outcomes—sexually violent behavior and prosocial bystander behavior. The aim of this paper was to present findings from the RCT with regard to 3 a priori hypotheses. First, we expected that GlobalConsent would *mitigate increases in sexually violent behavior* that may occur when men matriculate into university and interact with women with less parental supervision [41-43]. Second, we expected that GlobalConsent would *increase prosocial bystander behavior* relative to attention-control conditions. Third, we expected that GlobalConsent would influence these behavioral outcomes directly and indirectly through changes in knowledge, attitudinal, and affective secondary outcomes. The results presented here focused on the primary, unmediated behavioral outcomes.

## Methods

### Setting

Universities in Vietnam were suitable contexts for adapting RealConsent, given their similarities to universities in the United States. In Vietnam, undergraduate study is 4-6 years, with 2 foundational years and 2-4 years for specialization. Except for political education and national defense, universities design their curricular and extracurricular activities and maintain networks through the Ministry of Education and Training, professional associations, and the Youth Union, all pathways for the national scale-up of educational programs. The study sites were 2 universities located in Hanoi. One is a 120-year-old state school that trains 1000 students yearly in health professions. The other is a 34-year-old private university that trains 7000 students annually across diverse disciplines. Both universities provided letters of support for this study.

### Trial Design

The study design, detailed elsewhere [37], applied a double-blind, parallel intervention versus attention-control group design with balanced (1:1) randomization (Multimedia Appendix 1).

### Ethics Approval

Data were collected following established ethical guidelines for research on gender-based violence [37]. The institutional review boards of Emory University (IRB00099860) and Hanoi University of Public Health (017–384/DD-YTCC) reviewed and approved the study protocol, including the web-based consent forms used in the study.

### Sample Eligibility, Participation, and Retention

**Figure 1:**
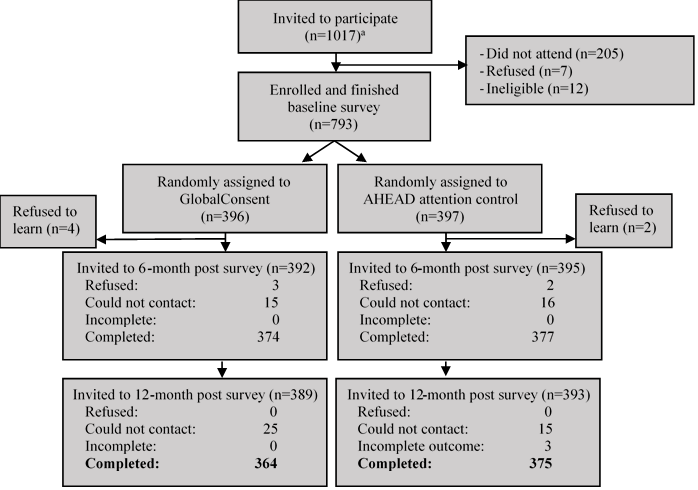
Flow diagram of sample enrollment, allocation, and follow-up randomized controlled trial of GlobalConsent. ^a^ Of the 1017 eligible men, 362 were from University 1 and 655 were from University 2. AHEAD: adolescent health education.

Eligible men for the study were heterosexual or bisexual, aged 18-24 years, and matriculating in September 2019 at either of the 2 study sites. To identify eligible men at university 1, overall, 56% (5/9) of the departments were included in the sample because all departments had an agreed threshold of ≥15 cismale students. For university 2, a total of 59% (13/22) of the departments having at least 20 cismale students were included. All first-year cismale students of the included departments were invited to participate (n=1017; Figure 1; power calculations are presented in the protocol paper by Yount et al [37]). Of these 1017 men, 205 (20.1%) did not attend the orientation, 7 (0.7%) declined, and 12 (1.2%) did not meet the inclusion criteria. Eligible, consenting men who completed an in-person, computer-assisted self-administered baseline survey at their home university in September 2019 (793/1017, 78%) were assigned a random number generated in Microsoft Excel (Microsoft Corp), sorted in ascending order. Of the 793 eligible men, the first 397 (50.1%) were assigned to the AHEAD attention-control group, and the remaining 396 (49.9%) men were assigned to the GlobalConsent group. The participants were blinded to these assignments because of the use of an attention-control condition, and the analysis team was blinded to these assignments throughout the analysis. Furthermore, 6 assigned participants (4 GlobalConsent and 2 AHEAD) declined to login to their assigned learning program. The participants were able to access their assigned programs from November 2019 to January 2020. Owing to the COVID-19 pandemic, posttest 1 (completed in April-May 2020, 3 months after intervention, and 6 months after baseline) and posttest 2 for trial endline (completed in October-November 2020, 9 months after intervention, and 12 months after baseline) were self-administered remotely via the web to ensure completion, regardless of students’ location of residence. Some internet disruptions were apparent, as the mean number of survey attempts was >1 in both groups and posttest rounds. However, the agreement across survey attempts between outcome-related responses exceeded 95%. In the GlobalConsent group, 94.4% (374/396) and 91.9% (364/396) students completed posttest 1 and 2, respectively. In the AHEAD group, 95% (377/397) and 94.5% (375/397) of students completed posttest 1 and 2, respectively. Reasons for high retention may have included a cultural tendency to adherence in Vietnam, a progressive compensation schedule for survey and program module completion, and text messages and emails to remind students to complete their studies [37].

### GlobalConsent Program

The adapted GlobalConsent program took into account qualitative findings [38,40] and feedback from focus group discussions with cismale students and university stakeholders on the RealConsent program and the adapted GlobalConsent storyboards before production (Multimedia Appendices 2 and 3). In total, 7 major adaptations have been observed. First, the local cismale characters were grounded in findings from the formative qualitative study, including the family and social contexts in which young men live in Vietnam. The male characters were adapted to evolve in contextualized ways, showing how their gender attitudes and awareness of sexual violence and response were changing. Thus, 5 cismale characters were created to reflect different initial masculinity typologies and different speeds and degrees of “masculinity development” over the program. These characters reflected (1) *positive masculinity* throughout; (2) *somewhat positive masculinity* throughout; (3) *somewhat more traditional masculinity* initially and becoming more aware earlier in the program (modules 2-3), as reflected in verbal tone, language, and marked behavior change; (4) *more traditional masculinity* initially and struggling but becoming more aware, as reflected in a scene in a karaoke bar when he returns to a hotel with drunk young woman and decides not to have sex; and (5) *very traditional masculinity* initially using inappropriate behavior, sanctioned by friends, and becoming more aware of modules 4-5, again as reflected in tone, language, and behavior.

Second, the cisfemale partners of these cismale characters were added because sexual coercion and violence in dating relationships emerged as salient in the formative qualitative findings. Third, “coach-talk” segments for reinforced learning were removed, as they relied on English vernacular, were misunderstood by Vietnamese stakeholders, and caused confusion about key messages. Fourth, “key questions” and “takeaways” were added to specific segments and scenarios, as these forms of reinforced learning were more familiar in Vietnam and helped to reduce misinterpretation of key messages. Fifth, content related to knowledge, attitudinal, and affective pathways in the theory of change was adapted to be relevant for Vietnam, and definitions of sexual violence were expanded to include the full spectrum of sexually violent behaviors. Sixth, anonymized narratives from the qualitative interviews were incorporated into the serial drama script to ensure comprehension and cultural suitability. Finally, all scenarios were refilmed or reanimated to resonate with the learning style of the cismale students in Vietnam.

The final GlobalConsent program included six 30-minute modules, each ranging in the number of segments and types of activities, with diverse actors and languages suitable for students who self-identify as heterosexual or bisexual men in Vietnam. Modules—grounded in social cognitive theory, social norms theory, and a bystander framework [37]—covered 6 topics to address hypothesized knowledge, normative or attitudinal, and affective mediators of the program and behavioral outcomes. These topics included consent for sex, rape-myth beliefs and norms regarding gender roles, effective communication, alcohol and rape, victim empathy, and bystander intervention. Each module included didactic activities, interactivity, and episodes of an adapted serial drama that modeled positive behaviors with cismale peers and cisfemale sex partners. The program was formatted for delivery to the computers and smartphones. Participants were provided with unique login information to access the platform and could work at their own pace, could not skip segments, and were encouraged by email and text messages to complete all modules in 12 weeks. The learning app captured the number of times a student logged in to each module and the total minutes a student spent on each module.

### AHEAD Attention-Control Program

The team developed AHEAD, the attention-control program, from open-access content suitable for young people in Vietnam [37]. To approximate the format, intensity, and duration of GlobalConsent, AHEAD was developed as a web-delivered, multimedia program audio-narrated in Vietnamese with 6 learning modules that were 35-45 minutes long on brain development, nutrition, physical activity, substance use, sleep, and agency. Similar to GlobalConsent, participants could work at their own pace, could not skip segments, and were encouraged by email and text messages to complete all modules in 12 weeks. The use of an attention-control condition was justified because the original trial of RealConsent also used an attention-control condition and the team wanted the trial of GlobalConsent to include a similar comparator. In addition, the use of an attention-control condition accounted for the web-based mode of delivery in both study arms, so any effects of the content of GlobalConsent could be compared with those of a general *adolescent health education* program that did not focus on the theoretical knowledge, attitudinal, and affective pathways by which GlobalConsent was theorized to reduce men’s sexually violent behavior and increase their prosocial bystander behavior.

### Data

#### Primary Outcomes

The primary outcomes for this analysis were sexually violent behavior, measured using the Sexual Experiences Survey [44], and prosocial bystander behavior, measured using 4 items from an adapted Bystander Behavior Scale [45] (Table 1). The Sexual Experiences Survey asks about the perpetration of 7 acts of contact sexual violence, ranging from unwanted touching to forced penetration, using any of 7 physical or nonphysical tactics, such as holding someone down or threatening to end the relationship (35 items total). The Sexual Experiences Survey also asked about the perpetration of 10 acts of noncontact sexual violence, such as masturbating in front of someone when they did not agree. We captured *sexually violent behavior* based on the reported frequency (never, once, twice, and ≥3 times) of all 45 items in the prior 6 months. We captured *prosocial bystander behavior* based on the reported frequency (never, once, and ≥2 times) in the prior 12 months of 4 acts, such as “I have talked with someone about sexual or dating violence as an issue for our university.” The frequency of missingness was 0.1%-0.7% across all items, and these responses were recoded as 0, “not reported.” We created kernel density plots of the summative scores for each primary outcome (Multimedia Appendix 4). On the basis of their distributions, we captured 3 measurement scales for each behavioral outcome to assess whether the GlobalConsent program had linear or threshold effects. These measurement scales were any reported act (1 or more vs 0 or none reported), many reported acts (3 or more vs 0-2 or none reported), and the number of reported acts (Table 1).

**Table 1:** Primary reported behavioral outcomes in the randomized controlled trial of GlobalConsent.

Outcomes	Instrument	Response options	Sample size, n	Example item	“Any act”	“Many acts”	Sum
Sexually violent behavior, past 6 months	Sexual Experiences Survey [44]	0, Never 1, Once 2, Twice 3, ≥3 times	45	I took photos or videotapes of someone while they were undressing, were nude, or were having sex, when they did not agree to it.	1, Any act 0, No act^a^	1, 3 or more acts 0, 0-2 acts^a^	0-135
Prosocial bystander behavior, past 12 months	Bystander Behavior Scale [45]	0, Never 1, Once 2, ≥2 times	4	I have encouraged others to learn more and get involved in preventing sexual or dating violence.	1, Any act 0, No act^a^	1, 3 or more acts 0, 0-2 acts^a^	0-8

^a^Missing responses for the number of acts were coded 0.

#### Exposures

Participants were assigned randomly to either the GlobalConsent treatment (=1) or the AHEAD attention-control program (=0). Time was coded 0 for baseline and 1 for subsequent time points. A time-by-treatment arm interaction term was created to assess the difference-in-difference (DID) for GlobalConsent versus AHEAD at “endline” (posttest 1 and 2 combined) versus baseline.

#### Covariates

Covariates included age in years, program of study (health sciences, others), relationship history (ever, never), living situation (parents, other relatives, dormitory, off campus, and other), ethnicity (majority, minority), sexual orientation (heterosexual, bisexual), religion (any, none), and residence in Hanoi for at least 1 year before baseline (yes, no). In addition, past sexual history (ever, never), sexual preference (women only, others), gender identity, and expression (very feminine=1 to very masculine=7) were collected at the posttest 1. At posttest 2, an adapted Vietnam Prevalence Study on Child Maltreatment [46] scale measured maltreatment before the age of 18 years with 28 items capturing any exposure to behaviors by adults in 5 domains: physical, sexual, and emotional abuse as well as physical and emotional neglect. Dichotomous (any, none) variables were created for each domain of child maltreatment. Any exposure (yes, no) to web-based sexually explicit material in the past 6 months was measured for 5 domains (textual, partial nudity, full nudity, nonviolent sexual acts, and violent sexual acts) [47].

#### Statistical Analysis

First, we conducted descriptive analyses of the sample overall and by study arm to assess qualitative balance across groups. Second, we assessed levels of sexually violent behavior and prosocial intervening behavior at each study wave by study arm. Finally, we conducted DID modeling to estimate the odds ratios (ORs), incidence rate ratios, and 95% CIs for sexually violent behavior and prosocial bystander behavior at endline (posttest 1 and 2 combined) versus baseline. For binary outcomes, we performed logistic regression with the assigned treatment arm, time, and treatment-by-time interaction (DID variable) as predictors. For count outcomes, we performed a negative binomial regression, which accounted for overdispersion in the outcome. To probe significant treatment-by-time interaction or DID effects, we computed and compared the OR and incidence rate ratio of the occurrence of the behavior separately for each group. All analyses were performed using Stata (version 16; StataCorp [48]).

### Role of the Funding Source

The funder of the study had no role in study design, data collection, analysis, interpretation, or writing of the report. All authors had full access to all study data and were responsible for the decision to submit this paper for publication.

## Results

### Sample Characteristics

The characteristics of men across the groups were similar at baseline and follow-up waves (Tables 2-4). At baseline, men were aged 18 years, on average, and a majority were attending university 2, majoring in nonhealth fields, of Kinh ethnicity, heterosexual, nonreligious, and had never been in a dating or sexual relationship. The living arrangements and residential location of men were similarly distributed across the groups (Table 2). After baseline, high percentages of men in both the GlobalConsent and AHEAD groups, respectively, logged into the program (372/396, 93.9% vs 386/397, 97.2%), completed at least 1 module (361/396, 91.2% vs 374/397, 94.2%), and completed all modules (355/396, 89.6% vs 362/397, 91.2%).

At posttest 1, most men in both groups had never had sex and sexually preferred only women (Table 3). In addition, reported gender identities and gender presentations were predominantly masculine, with average scores ≥6.0. About three-fourths (569/751, 75.8%) of men had ever been exposed to textual sexually explicit material or “top nudity.” Almost two-thirds (490/751, 65.2%) had ever been exposed to “full nudity” or nonviolent sexual acts. Approximately 40.9% (302/739; ie, about 4 in 10) had ever been exposed to sexually explicit material showing violent sexual acts against women.

Reported experiences of child maltreatment, by type, were similar in the GlobalConsent and AHEAD groups (Table 4). More than half (401/735, 54.6%) of the men reported experiencing emotional abuse in childhood, and about 1 in 5 reported experiencing emotional neglect in childhood. Almost half (346/736, 47%) of the men reported experiencing physical abuse in childhood and ≤5% reported experiencing physical neglect in childhood. More than 1 in 10 men reported experiencing sexual abuse during childhood. High levels of childhood maltreatment are consistent with 2014 population-based national data from secondary school students. Except for emotional neglect, which is higher, the prevalence estimates for childhood maltreatment in our sample are lower than the national average [46].

**Table 2:** Baseline Characteristics of men matriculating in 2 universities in Vietnam in September 2019, overall and by groups randomized to GlobalConsent treatment and adolescent health education (AHEAD) attention-control conditions.

Covariates measured at baseline		GlobalConsent (n=396)^a^	AHEAD (n=397)	Total (n=793)^a^
**University, n (%)**				
	University 1	172 (43.4)	173 (43.6)	345 (43.5)
	University 2	224 (56.6)	224 (56.4)	448 (56.5)
**Major, n (%)**				
	Health	185 (46.7)	181 (45.6)	366 (46.2)
	Others	211 (53.3)	216 (54.4)	427 (53.9)
Age (years), mean (SD)		18.1 (0.4)	18.1 (0.3)	18.1 (0.4)
**Ethnicity, n (%)**				
	Majority (Kinh)	377 (96.2)	379 (95.5)	756 (95.8)
	Minority	15 (3.8)	18 (4.5)	33 (4.2)
**Sexual orientation, n (%)**				
	Heterosexual	378 (95.5)	381 (96)	759 (95.7)
	Bisexual	18 (4.6)	16 (4)	34 (4.3)
**Religion, n (%)**				
	Any	62 (15.7)	73 (18.4)	135 (17)
	None	334 (84.3)	324 (81.6)	658 (83)
**Relationship status, n (%)**				
	Ever in a relationship	179 (45.2)	187 (47.1)	366 (46.2)
	Never in a relationship	217 (54.8)	210 (52.9)	427 (53.9)
**Living situation, n (%)**				
	With parents	233 (33.6)	119 (30)	252 (31.8)
	With other relatives	54 (13.6)	52 (13)	106 (13.4)
	Dormitory or on campus	52 (13.1)	68 (17.1)	120 (15.1)
	Off-campus alone or with nonrelatives	145 (36.6)	138 (34.8)	283 (35.7)
	Other do not know	12 (3)	20 (5)	32 (4)
**Lived in Hanoi at least 1 year, n (%)**				
	Yes	203 (51.3)	183 (46.1)	386 (48.7)
	No	193 (48.7)	214 (53.9)	407 (51.3)

^a^*Missing at baseline*: ethnicity 4 GlobalConsent.

**Table 3:** Characteristics measured at posttest 1 (6-months after baseline) of men matriculating in 2 universities in Vietnam in September 2019, overall and by groups randomized to GlobalConsent treatment and adolescent health education (AHEAD) attention-control conditions.

Covariates measured at posttest 1 (6 months after baseline)		GlobalConsent (n=374)^a^	AHEAD (n=377)^a^	Total (n=751)^a^
Survey attempts, mean (SD)		1.6 (1.9)	1.4 (0.8)	1.5 (1.5)
**Ever had sex, n (%)**				
	Yes	66 (18.1)	80 (22.3)	146 (20.2)
	Never	299 (81.9)	279 (77.7)	578 (79.8)
**Sexual preference, n (%)**				
	Only women	297 (81.4)	303 (84.4)	600 (82.9)
	Others or do not know	68 (18.6)	56 (15.6)	124 (17.1)
Gender identity (very feminine=1 to very masculine=7), mean (SD)		6.1 (0.9)	6.2 (0.9)	6.2 (0.9)
Gender presentation (very feminine=1 to very masculine=7), mean (SD)		6.0 (0.9)	6.0 (0.9)	6.0 (0.9)
**Exposure to SEM^b^: textual, n (%)**				
	Yes	286 (76.5)	297 (78.8)	583 (77.6)
	No	88 (23.5)	80 (21.2)	168 (22.4)
**Exposure to SEM: top nudity, n (%)**				
	Yes	279 (74.6)	290 (76.9)	569 (75.8)
	No	95 (25.4)	87 (23.1)	182 (24.2)
**Exposure to SEM: full nudity, n (%)**				
	Yes	241 ( 64.4)	249 (66.1)	490 (65.2)
	No	133 (35.6)	128 (34)	261 (34.8)
**Exposure to SEM: nonviolent sex, n (%)**				
	Yes	234 (62.8)	244 (64.7)	480 (63.9)
	No	139 (37.2)	133 (35.8)	271 (36.1)
**Exposure to SEM: violent sex, n (%)**				
	Yes	143 (38.2)	159 (42.2)	302 (40.2)
	No	231 (61.8)	218 (57.8)	449 (59.8)
Lost to follow-up at posttest 1, n (%)		22 (5.6)	20 (5)	42 (5.3)

^a^*Missing at posttest 1*: ever had sex 9 GlobalConent (GC), 18 AHEAD; sexual preference 9 GC, 18 AHEAD; gender identity 11 GC, 18 AHEAD; gender presentation 9 GC, 18 AHEAD.

^b^SEM: sexually explicit material.

**Table 4:** Characteristics measured at posttest 2 (12-months after baseline) of men matriculating in 2 universities in Vietnam in September 2019, overall and by groups randomized to GlobalConsent treatment and adolescent health education (AHEAD) attention-control conditions.

Covariates measured at posttest 2 (12 months after baseline)			GlobalConsent (n=364)^a^		AHEAD (n=375)^a^		Total (n=739)^a^
**Child maltreatment: emotional abuse, n (%)**							
	Yes	188 (51.9)		213 (56.6)		401 (54.6)	
	No	173 (48.1)		161 (43.1)		334 (45.4)	
**Child maltreatment: emotional neglect, n (%)**							
	Yes	75 (20.7)		65 (17.4)		140 (19)	
	No	287 (79.3)		309 (82.6)		596 (81)	
**Child maltreatment: physical abuse, n (%)**							
	Yes	171 (47.2)		175 (46.8)		346 (47)	
	No	191 (52.8)		199 (53.2)		390 (53)	
**Child maltreatment: physical neglect, n (%)**							
	Yes	9 (2.5)		19 (5.1)		28 (3.8)	
	No	353 (97.5)		355 (94.9)		708 (96.2)	
**Child maltreatment: sexual abuse, n (%)**							
	Yes	55 (15.2)		48 (12.8)		103 (14)	
	No	307 (84.8)		326 (87.2)		633 (86)	
Lost to follow-up at posttest 2, from baseline n (%)			32 (8.1)		22 (5.5)		54 (6.8)

^a^*Missing at posttest 2*: physical abuse, emotional abuse, sexual abuse, physical neglect, and emotional neglect same 2 in GlobalConsent and same 1 in AHEAD.

### Sexually Violent Behavior and Prosocial Intervening Behavior

Reported rates of any sexually violent behavior were 31.1% (123/396), 21.4% (80/374), and 31.9% (116/364) across survey waves in the GlobalConsent group and 25.9% (103/397), 26.3% (99/377), and 31.5% (118/375) across waves in the AHEAD group (Table 5). Rates of “high” sexually violent behavior (>2 acts) were 21% (83/396), 13.6% (51/374), and 24.7% (90/364) across waves in the GlobalConsent group and 17.1% (68/397), 18% (68/377), and 23.2% (87/375) across waves in the AHEAD group. The mean count of sexually violent acts was 3.2, 2.2, and 3.1 across waves in the GlobalConsent group and 2.4, 3.6, and 3.2 across waves in the AHEAD group.

**Table 5:** Sexually violent behavior and prosocial bystander behavior at baseline, posttest 1 (6 months after baseline), and posttest 2 (12 months after baseline) of men matriculating at 2 universities in Vietnam in September 2019 and randomized to receive the GlobalConsent program or the adolescent health education (AHEAD) attention-control program.

Outcomes		Baseline		Posttest 1			Posttest 2	
		AHEAD (n=397)	GlobalConsent (n=396)	AHEAD (n=377)	GlobalConsent (n=374)	AHEAD (n=375)		GlobalConsent (n=364)
**Sexually violent behavior**								
	Yes any, n (%)	103 (25.9)	123 (31.1)	99 (26.3)	80 (21.4)	118 (31.5)		116 (31.9)
	Yes, high (>2 acts), n (%)	68 (17.1)	83 (21)	68 (18)	51 (13.6)	87 (23.2)		90 (24.7)
	Count, mean (SE); range	2.4 (9.7); 0-111	3.2 (11.6); 0-96	3.55 (12.1); 0-112	2.24 (8.1); 0-70	3.2 (9.9); 0-79		3.1 (11.7); 0-135
**Prosocial bystander behavior**								
	Yes any, n (%)	258 (65)	258 (65.2)	N/A^a^	N/A	170 (45.3)		203 (55.8)
	Yes high (>2 acts), n (%)	141 (35.5)	163 (41.2)	N/A	N/A	108 (28.8)		138 (37.9)
	Count, mean (SE); range	2.2 (0.1); 0-8	2.4 (0.1); 0-8	N/A	N/A	1.7 (0.1); 0-8		2.3 (0.1); 0-8

^a^N/A: not applicable (as the 12-month window of observation at posttest 1 overlaps with the 12-month window of observation at baseline).

Reported rates of any prosocial bystander behavior were 65.2% (258/396) and 55.8% (203/364) across survey waves in the GlobalConsent group and 65% (258/397) and 45.3% (170/375) across waves in the AHEAD group (Table 5). Rates of “high” prosocial bystander behavior (>2 acts) were 41.2% (163/396) and 37.9% (138/364) across waves in the GlobalConsent group and were 35.5% (141/397) and 28.8% (108/375) across waves in the AHEAD group. The mean counts of prosocial bystander acts were 2.4 and 2.3 across waves in the GlobalConsent group and were 2.2 and 1.7 across waves in the AHEAD group.

### Effects of GlobalConsent on Behavioral Outcomes

Significant interaction effects in the DID models suggested favorable impacts of GlobalConsent relative to AHEAD on sexually violent behavior (>2 acts; OR 0.5, 95% CI 0.3-0.7; *P*=.001) and prosocial bystander behavior (any act; OR 1.5, 95% CI 1.0-2.3; *P*=.05; Table 6). Marginally significant interaction effects in DID models for any sexually violent behavior and the count for prosocial bystander acts suggested a consistent, favorable impact of GlobalConsent relative to AHEAD across the measurement scales of these 2 behavioral outcomes.

**Table 6:** Difference-in-difference odds ratios (ORs) for the logistic model or incidence rate ratios (IRRs) for the negative binomial model, comparing endline (posttest 1 and 2 combined) versus baseline for 730 university men in the GlobalConsent versus adolescent health education groups.

Outcomes			OR IRR (95% CI)		*P* value
**Sexually violent behavior**					
	Yes, any act	0.71 (0.50-1.00)		.05	
	Yes, >2 acts	0.47 (0.31-0.72)		.001	
	Count	0.65 (0.37-1.14)		.13	
**Prosocial bystander behavior**					
	Yes, any act	1.51 (1.00-2.27)		.05	
	Yes, >2 acts	1.19 (0.78-1.81)		.42	
	Count	1.27 (1.00-1.60)		.05	

To probe the interaction effects further, we computed the ORs of sexually violent behavior (>2 acts) and prosocial bystander behavior (any act) at endline (posttest 1 and 2 combined) versus baseline separately for the GlobalConsent and AHEAD groups. Among the GlobalConsent participants, the odds of perpetrating >2 acts of sexual violence at the endline were 1.3 times the odds at baseline. For the AHEAD group, the odds of perpetrating >2 acts of sexual violence at the endline were 2.7 times the odds at baseline. Among the GlobalConsent participants, the odds of any prosocial behavior at endline were 0.7 times the odds at baseline. For the AHEAD group, the odds of any prosocial behavior at posttest were 0.5 times the odds at baseline.

## Discussion

### Principal Findings

This randomized controlled trial recruited 793 university men in Vietnam to test the efficacy of GlobalConsent, a culturally tailored adaptation of an efficacious, theoretically grounded web-based sexual violence prevention program originally designed for US college men [23]. In this study, compared with men in the attention-control group, men in the GlobalConsent group had lower odds of engaging in sexually violent behavior at the endline than at the baseline. In addition, compared with men in the AHEAD group, men in the GlobalConsent group had higher odds of any prosocial bystander behavior at the endline than at baseline. The favorable impacts of GlobalConsent versus AHEAD were consistent across the measurement scales of these 2 behavioral outcomes. Such behavior changes are notable, as this is the first web-delivered sexual violence prevention program for cismale college students implemented in a middle-income setting. Most sexual violence prevention programs globally have measured changes in reported behavioral *intentions* rather than in reported *behavior* [49].

### Study Limitations and Strengths

This study had some limitations. First, the study sites were limited to 2 urban universities to accommodate program adaptation, production, and testing. Therefore, the findings cannot be generalized to all university men in Vietnam. However, the 2 study universities represented public and private institutions of higher education, diverse faculties of study, and diverse student bodies from urban and provincial Vietnam. Second, behavioral outcomes were self-reported and may have been subject to social desirability bias. To mitigate this limitation, the team relied on a validated instrument to measure sexual violence and self-administration, which enhances privacy and may enhance honest disclosures. Third, we did not interview women on campus about changes in behavior, as reported by men, owing to resource constraints and the ethical and logistical challenges of linking women’s reports of men’s behavior at the individual level. Fourth, some nonsignificant differences in behavior and attenuation of behavior change at 12 months suggest the need for booster training after the 3-hour program period. Fifth, the 12-month follow-up period allowed for a short-term impact assessment of GlobalConsent. However, the follow-up period in this study exceeded that in most similar studies. Long-term follow-up of participants would be beneficial to assess the impact of GlobalConsent on behavioral outcomes throughout men’s time at university and beyond. Sixth, similar to other web-based interventions, the content of GlobalConsent may become outdated, and updating or future adaptations to GlobalConsent may incur new costs. To mitigate this limitation, the team adapted a program script and content to ensure maximum durability. Seventh, participants in the GlobalConsent group may have become unblinded to their treatment assignment. Although a possibility, having an attention-control condition in which adolescent-focused health content is provided should reduce the likelihood of unblinding because health-related content is provided in both arms, and participants were not informed about the specific theoretical knowledge-, attitudinal-, and empathy-related pathways by which the GlobalConsent programing was expected to operate. Finally, some loss to follow-up was observed in both study arms. However, in absolute terms and relative to similar intervention studies [20-23], attrition from our study was very low and similarly low for the treatment and control arms. In addition, the baseline characteristics of those who were lost to follow-up were similar across the treatment and control arms. These findings reduce concerns that men who were sexually violent after baseline attrited more often from the treatment group than the control group; however, this possibility cannot be ruled out. Finally, as the DID method focused on changes, it accommodated the baseline differences in both main outcomes between the 2 arms.

Recognizing these potential limitations, the strengths of this study were many. They included a randomized controlled design, customized attention-control condition, web-based delivery of GlobalConsent and attention-control programs to computers and smartphones, a large sample of participating university men, outstanding participation and retention, and refined measurement of primary behavioral outcomes and secondary (mediating) outcomes at 3 months and 9 months after intervention. Ours is among the few RCTs in the field, especially in LMICs, to assess impacts on 2 behaviors and to assess the impact on behavior at 2 time points after intervention.

### Implications for Research and Public Health Practice

This study fills critical knowledge gaps on preventing sexual violence globally. In LMICs, interventions to prevent sexual violence in young men are rare [19]. This project adds to the limited evidence from LMICs on efficacious strategies to prevent sexual violence and promote prosocial bystander behaviors in university men. As GlobalConsent is an adaptation of RealConsent, this study adds to the limited evidence on the replication of efficacious sexual violence prevention interventions [50]. The review of bystander interventions by Mujal et al [20] identified 2 programs with evidence of replication; both were group-delivered and relied on presentations and discussions—techniques that can be resource- and cost-intensive. This study provides empirical support for the efficacy of a web-based edutainment approach for sexual violence prevention delivered to the computers and smartphones of students on college campuses, which may be more appealing and feasible in resource-constrained LMICs [51,52]. Finally, globally, most sexual violence prevention interventions have used in-person, small-group formats, with limited reach, standardization, and impact [50,53]. The web-based platform and GlobalConsent delivered to computers and smartphones make wide-scale implementation with higher fidelity more feasible than in-person programs. A large-scale implementation study of GlobalConsent in universities across Vietnam would be a robust next step to assess its effectiveness, and information about barriers and facilitators to university delivery of this intervention would be critical for moving GlobalConsent from research to practice.

This study reveals the value of using a systematic process to adapt evidence-based programs before they are delivered in new settings and populations. The process [36] used here allowed for the preservation of core content responsible for the efficacy of RealConsent and flexibility to add and to tailor other elements for university populations in Vietnam. Although the results here cannot confirm that GlobalConsent was an optimal adaptation to RealConsent, findings from the trial still suggest that following a systematic process to adapt an EBI may yield an adapted program that is efficacious cross culturally. This study demonstrates that GlobalConsent was able to reduce the risk of sexual violence and to promote prosocial bystander behavior in this LMIC. The potentially smaller effects of a web-based intervention are counterbalanced by GlobalConsent’s highly standardized delivery relative to in-person, small-group formats; therefore, the consistent delivery and scalability of GlobalConsent to the national level is likely to be high. Research to adapt and test GlobalConsent in other LMICs is warranted.

### Conclusions

Given the high rates of sexual violence against women, growing numbers of young men and women attaining postsecondary education, and rapid increases in access to the internet and smartphones globally, universities are ideal settings to provide novel sexual violence prevention programs during this critical developmental window, when young men are leaving home and are at increasing risk of sexually violent behavior. Evidence-based sexual violence prevention programs such as GlobalConsent, which are cost-effective, easily implemented by universities, and appealing to a diverse student population, are needed globally. Reviews of sexual violence prevention programs have described the importance of bystander approaches and engaging men as women’s allies in preventing sexual violence. Equally important is the ability of such efficacious programs to reach large populations to expand their impact. This study demonstrates that theoretically grounded, web-based edutainment with gender-specific content, customized didactic and interactive behavior change techniques, and an adapted serial drama make GlobalConsent potentially scalable in Vietnam and adaptable to other LMICs, where efficacious sexual violence prevention programs are needed.

## Appendix

Multimedia Appendix 1 CONSORT-eHEALTH checklist (V 1.6.1).

Multimedia Appendix 2 GlobalConsent and adolescent health education (AHEAD) program screenshots.

Multimedia Appendix 3 Documentation of adaptation of RealConsent to create GlobalConsent.

Multimedia Appendix 4 Kernel density plots of behavioral outcomes measured as counts.

## Abbreviations

AHEAD: adolescent health education
DID: difference-in-difference
EBI: evidence-based intervention
LMIC: lower- and middle-income country
OR: odds ratio
RCT: randomized controlled trial
