# Supplementary material for: Impacts of GlobalConsent, a Web-Based Social Norms Edutainment Program, on Sexually Violent Behavior and Bystander Behavior Among University Men in Vietnam: Randomized Controlled Trial
Source: JMIR Public Health Surveill. 2023 Jan 27;9:e35116. doi: 10.2196/35116 (PMC9919511; doi:10.2196/35116)
Supplement: Multimedia Appendix 1 [file publichealth_v9i1e35116_app1.pdf]

CONSORT-EHEALTH (V 1.6.1) - Submission/Publication Form

The CONSORT-EHEALTH checklist is intended for authors of randomized trials evaluating web-based and Internet-based applications/interventions, including mobile interventions, electronic games (incl multiplayer games), social media, certain telehealth applications, and other interactive and/or networked electronic applications. Some of the items (e.g. all subitems under item 5 - description of the intervention) may also be applicable for other study designs.

The goal of the CONSORT EHEALTH checklist and guideline is to be
a) a guide for reporting for authors of RCTs,
b) to form a basis for appraisal of an ehealth trial (in terms of validity)

CONSORT-EHEALTH items/subitems are MANDATORY reporting items for studies published in the Journal of Medical Internet Research and other journals / scientific societies endorsing the checklist.

Items numbered 1., 2., 3., 4a., 4b etc are original CONSORT or CONSORT-NPT (non-pharmacologic treatment) items.
Items with Roman numerals (i., ii, iii, iv etc.) are CONSORT-EHEALTH extensions/clarifications.

As the CONSORT-EHEALTH checklist is still considered in a formative stage, we would ask that you also RATE ON A SCALE OF 1-5 how important/useful you feel each item is FOR THE PURPOSE OF THE CHECKLIST and reporting guideline (optional).

Mandatory reporting items are marked with a red *.
In the textboxes, either copy & paste the relevant sections from your manuscript into this form - please include any quotes from your manuscript in QUOTATION MARKS,
or answer directly by providing additional information not in the manuscript, or elaborating on why the item was not relevant for this study.

YOUR ANSWERS WILL BE PUBLISHED AS A SUPPLEMENTARY FILE TO YOUR PUBLICATION IN JMIR AND ARE CONSIDERED PART OF YOUR PUBLICATION (IF ACCEPTED).
Please fill in these questions diligently. Information will not be copyedited, so please use proper spelling and grammar, use correct capitalization, and avoid abbreviations.

DO NOT FORGET TO SAVE AS PDF _AND_ CLICK THE SUBMIT BUTTON SO YOUR ANSWERS ARE IN OUR DATABASE !!!

Citation Suggestion (if you append the pdf as Appendix we suggest to cite this paper in the caption):
Eysenbach G, CONSORT-EHEALTH Group
CONSORT-EHEALTH: Improving and Standardizing Evaluation Reports of Web-based and

Mobile Health Interventions
J Med Internet Res 2011;13(4):e126
URL: http://www.jmir.org/2011/4/e126/
doi: 10.2196/jmir.1923
PMID: 22209829

ibergenfeld@gmail.com (not shared) Switch account

Draft not saved

* Required

Your name *

First Last

Irina Bergenfeld

Primary Affiliation (short), City, Country *

University of Toronto, Toronto, Canada

Emory University

Your e-mail address *

abc@gmail.com

ibergen@emory.edu

Title of your manuscript *

Provide the (draft) title of your manuscript.

Impacts of A Web-Based Social-Norms Edutainment Program on Sexually Violent Behavior and Bystander Behavior among University Men in Vietnam: The GlobalConsent Randomized Controlled Trial

Name of your App/Software/Intervention *

If there is a short and a long/alternate name, write the short name first and add the long name in brackets.

GlobalConsent

Evaluated Version (if any)

e.g. "V1", "Release 2017-03-01", "Version 2.0.27913"

Your answer

Language(s) *

What language is the intervention/app in? If multiple languages are available, separate by comma (e.g. "English, French")

Vietnamese

URL of your Intervention Website or App

e.g. a direct link to the mobile app on app in appstore (itunes, Google Play), or URL of the website. If the intervention is a DVD or hardware, you can also link to an Amazon page.

Your answer

URL of an image/screenshot (optional)

Your answer

Accessibility *

Can an enduser access the intervention presently?

- ○ access is free and open
- ◉ access only for special usergroups, not open
- ○ access is open to everyone, but requires payment/subscription/in-app purchases
- ○ app/intervention no longer accessible
- ○ Other:

Primary Medical Indication/Disease/Condition *

e.g. "Stress", "Diabetes", or define the target group in brackets after the condition, e.g. "Autism (Parents of children with)", "Alzheimers (Informal Caregivers of)"

Sexually violent behavior

Primary Outcomes measured in trial *

comma-separated list of primary outcomes reported in the trial

Sexually violent behavior, prosocial bystander l

Secondary/other outcomes

Are there any other outcomes the intervention is expected to affect?

Your answer

Recommended "Dose" *

What do the instructions for users say on how often the app should be used?

- ( ) Approximately Daily
- ( ) Approximately Weekly
- ( ) Approximately Monthly
- ( ) Approximately Yearly
- (x) "as needed"
- ( ) Other:

Approx. Percentage of Users (starters) still using the app as recommended after 3 months *

- ( ) unknown / not evaluated
- ( ) 0-10%
- ( ) 11-20%
- ( ) 21-30%
- ( ) 31-40%
- ( ) 41-50%
- ( ) 51-60%
- ( ) 61-70%
- ( ) 71%-80%
- ( ) 81-90%
- (x) 91-100%
- ( ) Other:

Overall, was the app/intervention effective? *

- ○ yes: all primary outcomes were significantly better in intervention group vs control
- ◉ partly: SOME primary outcomes were significantly better in intervention group vs control
- ○ no statistically significant difference between control and intervention
- ○ potentially harmful: control was significantly better than intervention in one or more outcomes
- ○ inconclusive: more research is needed
- ○ Other:

Article Preparation Status/Stage *

At which stage in your article preparation are you currently (at the time you fill in this form)

- ○ not submitted yet - in early draft status
- ◉ not submitted yet - in late draft status, just before submission
- ○ submitted to a journal but not reviewed yet
- ○ submitted to a journal and after receiving initial reviewer comments
- ○ submitted to a journal and accepted, but not published yet
- ○ published
- ○ Other:

Journal *

If you already know where you will submit this paper (or if it is already submitted), please provide the journal name (if it is not JMIR, provide the journal name under "other")

- ( ) not submitted yet / unclear where I will submit this
- (•) Journal of Medical Internet Research (JMIR)
- ( ) JMIR mHealth and UHealth
- ( ) JMIR Serious Games
- ( ) JMIR Mental Health
- ( ) JMIR Public Health
- ( ) JMIR Formative Research
- ( ) Other JMIR sister journal
- ( ) Other:

Is this a full powered effectiveness trial or a pilot/feasibility trial? *

- ( ) Pilot/feasibility
- (•) Fully powered

Manuscript tracking number *

If this is a JMIR submission, please provide the manuscript tracking number under "other" (The ms tracking number can be found in the submission acknowledgement email, or when you login as author in JMIR. If the paper is already published in JMIR, then the ms tracking number is the four-digit number at the end of the DOI, to be found at the bottom of each published article in JMIR)

- (•) no ms number (yet) / not (yet) submitted to / published in JMIR
- ( ) Other:

TITLE AND ABSTRACT

1a) TITLE: Identification as a randomized trial in the title

1a) Does your paper address CONSORT item 1a? *

I.e does the title contain the phrase "Randomized Controlled Trial"? (if not, explain the reason under "other")

- (x) yes
- ( ) Other:

1a-i) Identify the mode of delivery in the title

Identify the mode of delivery. Preferably use “web-based” and/or “mobile” and/or “electronic game” in the title. Avoid ambiguous terms like “online”, “virtual”, “interactive”. Use “Internet-based” only if Intervention includes non-web-based Internet components (e.g. email), use “computer-based” or “electronic” only if offline products are used. Use “virtual” only in the context of “virtual reality” (3-D worlds). Use “online” only in the context of “online support groups”. Complement or substitute product names with broader terms for the class of products (such as “mobile” or “smart phone” instead of “iphone”), especially if the application runs on different platforms.

	1	2	3	4	5	
subitem not at all important	( )	( )	( )	(x)	( )	essential

Clear selection

Does your paper address subitem 1a-i? *

Copy and paste relevant sections from manuscript title (include quotes in quotation marks "like this" to indicate direct quotes from your manuscript), or elaborate on this item by providing additional information not in the ms, or briefly explain why the item is not applicable/relevant for your study

Impacts of A Social-Norms Edutainment Program on Sexually Violent Behavior and Bystander Behavior among University Men in Vietnam: The GlobalConsent Randomized Controlled Trial

1a-ii) Non-web-based components or important co-interventions in title

Mention non-web-based components or important co-interventions in title, if any (e.g., “with telephone support”).

	1	2	3	4	5	
subitem not at all important	○	○	○	◉	○	essential

Clear selection

Does your paper address subitem 1a-ii?

Copy and paste relevant sections from manuscript title (include quotes in quotation marks "like this" to indicate direct quotes from your manuscript), or elaborate on this item by providing additional information not in the ms, or briefly explain why the item is not applicable/relevant for your study

There were no non-web-based interventions in the study

1a-iii) Primary condition or target group in the title

Mention primary condition or target group in the title, if any (e.g., “for children with Type I Diabetes”)
Example: A Web-based and Mobile Intervention with Telephone Support for Children with Type I Diabetes: Randomized Controlled Trial

	1	2	3	4	5	
subitem not at all important	○	○	○	○	◉	essential

Clear selection

Does your paper address subitem 1a-iii? *

Copy and paste relevant sections from manuscript title (include quotes in quotation marks "like this" to indicate direct quotes from your manuscript), or elaborate on this item by providing additional information not in the ms, or briefly explain why the item is not applicable/relevant for your study

Impacts of A Social-Norms Edutainment Program on Sexually Violent Behavior and Bystander Behavior among University Men in Vietnam: The GlobalConsent Randomized Controlled Trial

1b) ABSTRACT: Structured summary of trial design, methods, results, and conclusions

NPT extension: Description of experimental treatment, comparator, care providers, centers, and blinding status.

1b-i) Key features/functionalities/components of the intervention and comparator in the METHODS section of the ABSTRACT

Mention key features/functionalities/components of the intervention and comparator in the abstract. If possible, also mention theories and principles used for designing the site. Keep in mind the needs of systematic reviewers and indexers by including important synonyms. (Note: Only report in the abstract what the main paper is reporting. If this information is missing from the main body of text, consider adding it)

	1	2	3	4	5	
subitem not at all important	○	○	○	●	○	essential

Clear selection

Does your paper address subitem 1b-i? *

Copy and paste relevant sections from the manuscript abstract (include quotes in quotation marks "like this" to indicate direct quotes from your manuscript), or elaborate on this item by providing additional information not in the ms, or briefly explain why the item is not applicable/relevant for your study

Consenting heterosexual or bisexual men 18-24 years and matriculating in September 2019 (n=793) were enrolled and assigned randomly to GlobalConsent or an attention-control adolescent health education program (AHEAD). GlobalConsent is an adapted, theory-based, six-module web-based intervention with diverse behavioral-change techniques and a locally produced serial drama. AHEAD is a customized, six-module attention-control program on adolescent health. Both programs were delivered to computers and smartphones over 12 weeks.

1b-ii) Level of human involvement in the METHODS section of the ABSTRACT

Clarify the level of human involvement in the abstract, e.g., use phrases like “fully automated” vs. “therapist/nurse/care provider/physician-assisted” (mention number and expertise of providers involved, if any). (Note: Only report in the abstract what the main paper is reporting. If this information is missing from the main body of text, consider adding it)

	1	2	3	4	5	
subitem not at all important	○	○	◉	○	○	essential

Clear selection

Does your paper address subitem 1b-ii?

Copy and paste relevant sections from the manuscript abstract (include quotes in quotation marks "like this" to indicate direct quotes from your manuscript), or elaborate on this item by providing additional information not in the ms, or briefly explain why the item is not applicable/relevant for your study

"Both programs were delivered to computers and smartphones over 12 weeks."

1b-iii) Open vs. closed, web-based (self-assessment) vs. face-to-face assessments in the METHODS section of the ABSTRACT

Mention how participants were recruited (online vs. offline), e.g., from an open access website or from a clinic or a closed online user group (closed usergroup trial), and clarify if this was a purely web-based trial, or there were face-to-face components (as part of the intervention or for assessment). Clearly say if outcomes were self-assessed through questionnaires (as common in web-based trials). Note: In traditional offline trials, an open trial (open-label trial) is a type of clinical trial in which both the researchers and participants know which treatment is being administered. To avoid confusion, use “blinded” or “unblinded” to indicated the level of blinding instead of “open”, as “open” in web-based trials usually refers to “open access” (i.e. participants can self-enrol). (Note: Only report in the abstract what the main paper is reporting. If this information is missing from the main body of text, consider adding it)

	1	2	3	4	5	
subitem not at all important	○	○	○	○	◉	essential

Clear selection

Does your paper address subitem 1b-iii?

Copy and paste relevant sections from the manuscript abstract (include quotes in quotation marks "like this" to indicate direct quotes from your manuscript), or elaborate on this item by providing additional information not in the ms, or briefly explain why the item is not applicable/relevant for your study

"We applied a double-blind, parallel intervention-versus-control-group design with 1:1 randomization in two universities. Consenting heterosexual or bisexual men 18-24 years and matriculating in September 2019 (n=793) were enrolled and assigned randomly to GlobalConsent or an attention-control adolescent health education program (AHEAD)."

"Self-reported sexually violent behaviors toward women in the prior six months and prosocial bystander behaviors in the prior year were measured at zero, six, and 12 months."

1b-iv) RESULTS section in abstract must contain use data

Report number of participants enrolled/assessed in each group, the use/uptake of the intervention (e.g., attrition/adherence metrics, use over time, number of logins etc.), in addition to primary/secondary outcomes. (Note: Only report in the abstract what the main paper is reporting. If this information is missing from the main body of text, consider adding it)

	1	2	3	4	5	
subitem not at all important	○	○	○	○	○	essential

Does your paper address subitem 1b-iv?

Copy and paste relevant sections from the manuscript abstract (include quotes in quotation marks "like this" to indicate direct quotes from your manuscript), or elaborate on this item by providing additional information not in the ms, or briefly explain why the item is not applicable/relevant for your study

"Over 91% of men in both study arms completed at least one program module, and over 88% completed all six modules. At baseline, notable percentages of men reported any sexually violent behavior (GlobalConsent: 31.06%, n=396, AHEAD: 25.94%, n=397) in the prior six months. At endline (posttests 1 and 2 combined) compared to baseline, men receiving GlobalConsent had 1.26 times the odds of reporting a high level (at least two acts) of sexually violent behavior, and 0.67 times the odds of reporting any bystander behavior. The corresponding odds ratios for the AHEAD peers were less favorable, 2.67 and 0.45."

1b-v) CONCLUSIONS/DISCUSSION in abstract for negative trials

Conclusions/Discussions in abstract for negative trials: Discuss the primary outcome - if the trial is negative (primary outcome not changed), and the intervention was not used, discuss whether negative results are attributable to lack of uptake and discuss reasons. (Note: Only report in the abstract what the main paper is reporting. If this information is missing from the main body of text, consider adding it)

	1	2	3	4	5	
subitem not at all important	○	○	○	◉	○	essential

Clear selection

Does your paper address subitem 1b-v?

Copy and paste relevant sections from the manuscript abstract (include quotes in quotation marks "like this" to indicate direct quotes from your manuscript), or elaborate on this item by providing additional information not in the ms, or briefly explain why the item is not applicable/relevant for your study

Our trial did not have negative results.

INTRODUCTION

2a) In INTRODUCTION: Scientific background and explanation of rationale

2a-i) Problem and the type of system/solution

Describe the problem and the type of system/solution that is object of the study: intended as stand-alone intervention vs. incorporated in broader health care program? Intended for a particular patient population? Goals of the intervention, e.g., being more cost-effective to other interventions, replace or complement other solutions? (Note: Details about the intervention are provided in "Methods" under 5)

	1	2	3	4	5	
subitem not at all important	○	○	○	●	○	essential

Clear selection

Does your paper address subitem 2a-i? *

Copy and paste relevant sections from the manuscript (include quotes in quotation marks "like this" to indicate direct quotes from your manuscript), or elaborate on this item by providing additional information not in the ms, or briefly explain why the item is not applicable/relevant for your study

Sexual violence is any sexual act committed against a person without freely given consent. Sexual violence ranges from unwanted non-contact sexual experiences to completed forced penetration [1]. Among sexually experienced women 15–19 years, forced sexual debut is common worldwide (14.9%), including in the Asia/Pacific (13.8%) [2],and women comprise 91% of victims [3]. The physical, psychological, and economic aftermath of sexual violence often is severe for victims [4, 5] and is costly for societies [6].
In Vietnam, sexual violence persists [7, 8] despite legal reforms to address it. Men may discount, excuse, or deny acts of sexual violence [9], and their reported rates of sexually violent behavior (0.2%) are lower than women's reported rates of victimization (12.0%) [10]. Thus, prevention with men is crucial to create environments where women's freedom from violence is possible [11]. Yet, young men often are difficult to reach [12] and may resist participation in prevention programs [13]. The behavior of bystanders, or witnesses of sexual violence, also may be gender-specific [14]. Thus, prevention with men that integrates a bystander framework and treats men as "allies" [15] may address attitudes and behaviors while decreasing resistance to participation [16, 17].

...we undertook a randomized controlled trial (RCT) to test, among men attending two universities in Vietnam, the impact of GlobalConsent, versus a customized health-education attention-control condition (AHEAD), on changes in two primary behavioral outcomes—sexually violent behavior and prosocial bystander behavior. We expected that GlobalConsent would mitigate increases in sexually violent behavior that may occur when men matriculate into university and interact with women with less parental supervision [41-43]. We also expected that GlobalConsent would increase prosocial bystander behavior relative to attention-control conditions. Lastly, we expected that GlobalConsent would influence these behavioral outcomes directly and indirectly through changes in knowledge, attitudinal, and affective secondary outcomes.

2a-ii) Scientific background, rationale: What is known about the (type of) system

Scientific background, rationale: What is known about the (type of) system that is the object of the study (be sure to discuss the use of similar systems for other conditions/diagnoses, if appropiate), motivation for the study, i.e. what are the reasons for and what is the context for this specific study, from which stakeholder viewpoint is the study performed, potential impact of findings [2]. Briefly justify the choice of the comparator.

	1	2	3	4	5	
subitem not at all important	○	○	○	○	◉	essential

Clear selection

Does your paper address subitem 2a-ii? *

Copy and paste relevant sections from the manuscript (include quotes in quotation marks "like this" to indicate direct quotes from your manuscript), or elaborate on this item by providing additional information not in the ms, or briefly explain why the item is not applicable/relevant for your study

"This study tested the impact of GlobalConsent (ClinicalTrials.gov Identifier NCT04147455) on sexually violent behavior and prosocial bystander behavior in university men in Vietnam. The team adapted GlobalConsent from RealConsent, an evidence-based web-delivered intervention tested among university men in the United States [23]. RealConsent is based on formative research [24], social cognitive theory [25], social norms theory [26], and the bystander education model [27]. Sexually violent behavior is theorized to arise from the interplay of socio-contextual factors, personal factors, and behavior. To operationalize this theory of change, programmatic features of RealConsent include gender-specific content [28-31] that resonates with the viewer [32] and diverse behavioral-change techniques [33], such as providing information and instruction on obtaining effective consent for sex and intervening safely, modeling communication and intervening behaviors, showing positive outcomes for getting consent and intervening plus negative outcomes for perpetrating and not intervening, and reinforcing with positive feedback [34]. RealConsent uses didactic presentation of material via integrated audio, video, and infographics; problem-based learning with interactivity and reinforced practice; short videos or animations to model behavior; and educational entertainment, starting and ending each module with a brief episode of a serial drama [23, 35].
RealConsent aimed to change two primary behaviors by changing seven cognitive, attitudinal, and affective mediators. The two primary behaviors were prosocial intervening behaviors, such as trying to stop a peer from being sexually coercive, and sexually violent behavior toward women. The seven mediators included knowledge about the elements of sexual consent, knowledge and skills to intervene safely, misperceptions in norms about sexual violence and rape, negative attitudes about date rape, positive masculinity, skills in sexual communication, and empathy for the victims of sexual violence."

"To adapt RealConsent for delivery in to a new context, we followed the Centers for Disease Control and Prevention's five-step process [36]. Step 1 assess involved assessing the target population, the evidence-based intervention (EBI) being considered for implementation, and the implementing agency's capacity to employ the intervention. Step 2 select entailed determining whether to adopt the EBI without adaptations, with adaptations, or to choose another EBI for adaptation. Step 3 prepare involved making the needed adaptations to the EBI while retaining core elements. Step 4 pilot entailed piloting the adapted intervention and developing a plan for implementation. We conducted qualitative research to implement Steps 1–4 [37], and those findings are presented elsewhere [38-40]. To complete Step 5 implement, we undertook a randomized controlled trial (RCT) to test, among men attending two universities in Vietnam, the impact of GlobalConsent, versus a customized health-education attention-control condition (AHEAD), on changes in two primary behavioral outcomes—sexually violent behavior and prosocial bystander behavior. We expected that GlobalConsent would mitigate increases in sexually violent behavior that may occur when men matriculate into university and interact with women with less parental supervision [41-43]. We also expected that GlobalConsent would increase prosocial bystander behavior relative to attention-control conditions. Lastly, we expected that GlobalConsent would influence these behavioral outcomes directly and indirectly through changes in knowledge, attitudinal, and affective secondary outcomes. The results presented here focused on the primary, unmediated behavioral outcomes."

2b) In INTRODUCTION: Specific objectives or hypotheses

Does your paper address CONSORT subitem 2b? *

Copy and paste relevant sections from the manuscript (include quotes in quotation marks "like this" to indicate direct quotes from your manuscript), or elaborate on this item by providing additional information not in the ms, or briefly explain why the item is not applicable/relevant for your study

"To complete Step 5 implement, we undertook a randomized controlled trial (RCT) to test, among men attending two universities in Vietnam, the impact of GlobalConsent, versus a customized health-education attention-control condition (AHEAD), on changes in two primary behavioral outcomes—sexually violent behavior and prosocial bystander behavior. We expected that GlobalConsent would mitigate increases in sexually violent behavior that may occur when men matriculate into university and interact with women with less parental supervision [41-43]. We also expected that GlobalConsent would increase prosocial bystander behavior relative to attention-control conditions. Lastly, we expected that GlobalConsent would influence these behavioral outcomes directly and indirectly through changes in knowledge, attitudinal, and affective secondary outcomes."

METHODS

3a) Description of trial design (such as parallel, factorial) including allocation ratio

Does your paper address CONSORT subitem 3a? *

Copy and paste relevant sections from the manuscript (include quotes in quotation marks "like this" to indicate direct quotes from your manuscript), or elaborate on this item by providing additional information not in the ms, or briefly explain why the item is not applicable/relevant for your study

"The study design, detailed elsewhere [37], applied a double-blind, parallel intervention-versus-attention-control group design with balanced (1:1) randomization."

3b) Important changes to methods after trial commencement (such as eligibility criteria), with reasons

Does your paper address CONSORT subitem 3b? *

Copy and paste relevant sections from the manuscript (include quotes in quotation marks "like this" to indicate direct quotes from your manuscript), or elaborate on this item by providing additional information not in the ms, or briefly explain why the item is not applicable/relevant for your study

"Due to the COVID-19 pandemic, post-test one (completed in April-May, 2020, three months after intervention and six months after baseline) and post-test two (completed in October-November, 2020, nine months after intervention and 12 months after baseline) were self-administered remotely via the web to ensure completion, regardless of students' location of residence. "

3b-i) Bug fixes, Downtimes, Content Changes

Bug fixes, Downtimes, Content Changes: ehealth systems are often dynamic systems. A description of changes to methods therefore also includes important changes made on the intervention or comparator during the trial (e.g., major bug fixes or changes in the functionality or content) (5-iii) and other "unexpected events" that may have influenced study design such as staff changes, system failures/downtimes, etc. [2].

	1	2	3	4	5	
subitem not at all important	○	●	○	○	○	essential

Clear selection

Does your paper address subitem 3b-i?

Copy and paste relevant sections from the manuscript (include quotes in quotation marks "like this" to indicate direct quotes from your manuscript), or elaborate on this item by providing additional information not in the ms, or briefly explain why the item is not applicable/relevant for your study

This item is not applicable

4a) Eligibility criteria for participants

Does your paper address CONSORT subitem 4a? *

Copy and paste relevant sections from the manuscript (include quotes in quotation marks "like this" to indicate direct quotes from your manuscript), or elaborate on this item by providing additional information not in the ms, or briefly explain why the item is not applicable/relevant for your study

"For University 1, all nine departments were included in the sample, because all departments had an agreed threshold of 15 or more male students. For University 2, 13 of 22 departments having at least 20 male students were included. All first-year male students of included departments were invited to participate (n=1,017; Figure 1). Of these men, 205 did not attend the orientation, seven declined, and 12 did not meet the inclusion criteria of being heterosexual or bisexual, 18-24 years, and matriculating in September 2019 to either study university. "

4a-i) Computer / Internet literacy

Computer / Internet literacy is often an implicit “de facto” eligibility criterion - this should be explicitly clarified.

	1	2	3	4	5	
subitem not at all important	○	○	●	○	○	essential

Clear selection

Does your paper address subitem 4a-i?

Copy and paste relevant sections from the manuscript (include quotes in quotation marks "like this" to indicate direct quotes from your manuscript), or elaborate on this item by providing additional information not in the ms, or briefly explain why the item is not applicable/relevant for your study

No. Since participants are university students, computer literacy is assumed.

4a-ii) Open vs. closed, web-based vs. face-to-face assessments:

Open vs. closed, web-based vs. face-to-face assessments: Mention how participants were recruited (online vs. offline), e.g., from an open access website or from a clinic, and clarify if this was a purely web-based trial, or there were face-to-face components (as part of the intervention or for assessment), i.e., to what degree got the study team to know the participant. In online-only trials, clarify if participants were quasi-anonymous and whether having multiple identities was possible or whether technical or logistical measures (e.g., cookies, email confirmation, phone calls) were used to detect/prevent these.

	1	2	3	4	5	
subitem not at all important	○	○	○	○	◉	essential

Clear selection

Does your paper address subitem 4a-ii? *

Copy and paste relevant sections from the manuscript (include quotes in quotation marks "like this" to indicate direct quotes from your manuscript), or elaborate on this item by providing additional information not in the ms, or briefly explain why the item is not applicable/relevant for your study

"Eligible, consenting men who completed an in-person, computer-assisted self-administered baseline survey in September 2019 (n=793) each were assigned a random number generated in excel, sorted in ascending order. The first 397 men were assigned to the AHEAD attention-control group, and the remaining men were assigned to the GlobalConsent group. Participants were blinded to these assignments because of our use of an attention-control condition, and the analysis team was blinded to these assignments throughout the analysis. Six assigned participants (four GlobalConsent, two AHEAD) declined to login to their assigned learning program. Participants were able to access their assigned program from November 2019 to January 2020. Due to the COVID-19 pandemic, post-test one (completed in April-May, 2020, three months after intervention and six months after baseline) and post-test two (completed in October-November, 2020, nine months after intervention and 12 months after baseline) were self-administered remotely via the web to ensure completion, regardless of students' location of residence."

4a-iii) Information giving during recruitment

Information given during recruitment. Specify how participants were briefed for recruitment and in the informed consent procedures (e.g., publish the informed consent documentation as appendix, see also item X26), as this information may have an effect on user self-selection, user expectation and may also bias results.

	1	2	3	4	5	
subitem not at all important	○	○	○	○	◉	essential

Clear selection

Does your paper address subitem 4a-iii?

Copy and paste relevant sections from the manuscript (include quotes in quotation marks "like this" to indicate direct quotes from your manuscript), or elaborate on this item by providing additional information not in the ms, or briefly explain why the item is not applicable/relevant for your study

"For University 1, all nine departments were included in the sample, because all departments had an agreed threshold of 15 or more male students. For University 2, 13 of 22 departments having at least 20 male students were included. All first-year male students of included departments were invited to participate (n=1,017; Figure 1). Of these men, 205 did not attend the orientation, seven declined, and 12 did not meet the inclusion criteria of being heterosexual or bisexual, 18-24 years, and matriculating in September 2019 to either study university"

"The Institutional Review Boards of Emory University (IRB00099860) and the Hanoi University of Public Health (017–384/DD-YTCC) reviewed and approved the study protocol, including the use of oral consent forms for adults for each type of interview."

4b) Settings and locations where the data were collected

Does your paper address CONSORT subitem 4b? *

Copy and paste relevant sections from the manuscript (include quotes in quotation marks "like this" to indicate direct quotes from your manuscript), or elaborate on this item by providing additional information not in the ms, or briefly explain why the item is not applicable/relevant for your study

"Universities in Vietnam were suitable contexts for adapting RealConsent, given similarities with US universities. In Vietnam, undergraduate study is 4–6 years, with two foundational years and 2–4 years for specialization. Except for political education and national defense, universities design their curricular and extra-curricular activities and maintain networks through the Ministry of Education and Training, professional associations, and the Youth Union, all pathways for national scale-up of educational programs. The study sites were two universities in Hanoi. One, a 100-year-old state school, trains 1,000 students yearly in the health professions. The other, a 24-year-old private university, trains 7,000 students yearly in diverse disciplines. Both universities provided letters of support for this study."
"Eligible, consenting men who completed an in-person, computer-assisted self-administered baseline survey at their home university in September 2019 (n=793) each were assigned a random number generated in excel, sorted in ascending order."
""Due to the COVID-19 pandemic, post-test one (completed in April-May, 2020, three months after intervention and six months after baseline) and post-test two (completed in October-November, 2020, nine months after intervention and 12 months after baseline) were self-administered remotely via the web to ensure completion, regardless of students' location of residence."

4b-i) Report if outcomes were (self-)assessed through online questionnaires

Clearly report if outcomes were (self-)assessed through online questionnaires (as common in web-based trials) or otherwise.

	1	2	3	4	5	
subitem not at all important	○	○	○	○	●	essential

Clear selection

Does your paper address subitem 4b-i? *

Copy and paste relevant sections from the manuscript (include quotes in quotation marks "like this" to indicate direct quotes from your manuscript), or elaborate on this item by providing additional information not in the ms, or briefly explain why the item is not applicable/relevant for your study

"Eligible, consenting men who completed an in-person, computer-assisted self-administered baseline survey at their home university in September 2019 (n=793) each were assigned a random number generated in excel, sorted in ascending order."

4b-ii) Report how institutional affiliations are displayed

Report how institutional affiliations are displayed to potential participants [on ehealth media], as affiliations with prestigious hospitals or universities may affect volunteer rates, use, and reactions with regards to an intervention.(Not a required item – describe only if this may bias results)

	1	2	3	4	5	
subitem not at all important	◉	○	○	○	○	essential

Clear selection

Does your paper address subitem 4b-ii?

Copy and paste relevant sections from the manuscript (include quotes in quotation marks "like this" to indicate direct quotes from your manuscript), or elaborate on this item by providing additional information not in the ms, or briefly explain why the item is not applicable/relevant for your study

This is not applicable to the study

5) The interventions for each group with sufficient details to allow replication, including how and when they were actually administered

5-i) Mention names, credential, affiliations of the developers, sponsors, and owners

Mention names, credential, affiliations of the developers, sponsors, and owners [6] (if authors/evaluators are owners or developer of the software, this needs to be declared in a “Conflict of interest” section or mentioned elsewhere in the manuscript).

	1	2	3	4	5	
subitem not at all important	○	○	○	◉	○	essential

Clear selection

Does your paper address subitem 5-i?

Copy and paste relevant sections from the manuscript (include quotes in quotation marks "like this" to indicate direct quotes from your manuscript), or elaborate on this item by providing additional information not in the ms, or briefly explain why the item is not applicable/relevant for your study

Your answer

5-ii) Describe the history/development process

Describe the history/development process of the application and previous formative evaluations (e.g., focus groups, usability testing), as these will have an impact on adoption/use rates and help with interpreting results.

	1	2	3	4	5	
subitem not at all important	○	○	○	◉	○	essential

Clear selection

Does your paper address subitem 5-ii?

Copy and paste relevant sections from the manuscript (include quotes in quotation marks "like this" to indicate direct quotes from your manuscript), or elaborate on this item by providing additional information not in the ms, or briefly explain why the item is not applicable/relevant for your study

"GlobalConsent Program
The adapted GlobalConsent program took into account qualitative findings [38, 40] and feedback from male students and university stakeholders on the RealConsent program and the adapted GlobalConsent storyboards before production (Supplemental File 1). Seven major adaptations were notable. First, the four main male characters of the serial drama were modified to highlight locally salient aspects of masculinity. One character represented 'positive' masculinity, one 'somewhat positive' masculinity, one 'somewhat patriarchal' masculinity but more aware starting in Module 4, and one 'more patriarchal' masculinity and struggling to become more aware at the end of program. Second, female partners of these male characters were added because sexual coercion and violence in dating relationships were salient in the qualitative findings. Third, 'coach-talk' segments for reinforced learning were removed, as they relied on English vernacular, were misunderstood by Vietnamese stakeholders, and caused confusion about key messages. Fourth, 'key questions' and 'takeaways' were added for specific segments and scenarios—as these forms of reinforced learning were more familiar in Vietnam and helped to reduce misinterpretation of key messages. Fifth, content related to knowledge, attitudinal, and affective pathways in the theory of change were adapted to be relevant for Vietnam, and definitions of sexual violence were expanded to include the full spectrum of sexually violent behaviors. Sixth, anonymized narratives from the qualitative interviews were incorporated into the serial-drama script to ensure comprehension and cultural suitability. Finally, all scenarios were re-filmed or re-animated to resonate with the learning style of male students in Vietnam.
The final GlobalConsent program included six, 30-minute modules, each ranging in the number of segments and types of activities, with diverse actors and language suitable for male students in Vietnam (Supplemental File 1). Modules—grounded in social cognitive theory, social norms theory and a bystander framework [37]—covered six topics to address hypothesized knowledge, normative/attitudinal, and affective mediators of the program and behavioral outcomes. These topics were consent for sex, rape-myth beliefs and norms about gender roles, effective communication, alcohol and rape, victim empathy, and bystander intervention. Each module included didactic activities, interactivity, and episodes of an adapted serial drama that modeled positive behaviors with male peers and female sex partners. The program was formatted for delivery to computers and smartphones. Participants could work at their own pace, could not skip segments, and were encouraged by email and text messages to complete all modules in 12 weeks.
AHEAD Attention-Control Program
The team developed Adolescent Health Education (AHEAD), the health-education, attention-control program, from open-access content suitable for young people in Vietnam [37]. To approximate the format, intensity, and duration of GlobalConsent, AHEAD was developed as a web-delivered, multimedia program audio-narrated in Vietnamese with six 35–45 minute learning modules on brain development, nutrition, physical activity, substance use, sleep, and agency. Like GlobalConsent, participants could work at their own pace, could not skip segments, and were encouraged by email and text messages to complete all modules in 12 weeks."

5-iii) Revisions and updating

Revisions and updating. Clearly mention the date and/or version number of the application/intervention (and comparator, if applicable) evaluated, or describe whether the intervention underwent major changes during the evaluation process, or whether the development and/or content was “frozen” during the trial. Describe dynamic components such as news feeds or changing content which may have an impact on the replicability of the intervention (for unexpected events see item 3b).

	1	2	3	4	5	
subitem not at all important	○	○	◉	○	○	essential

Clear selection

Does your paper address subitem 5-iii?

Copy and paste relevant sections from the manuscript (include quotes in quotation marks "like this" to indicate direct quotes from your manuscript), or elaborate on this item by providing additional information not in the ms, or briefly explain why the item is not applicable/relevant for your study

The intervention was not revised during the trial

5-iv) Quality assurance methods

Provide information on quality assurance methods to ensure accuracy and quality of information provided [1], if applicable.

	1	2	3	4	5	
subitem not at all important	○	○	◉	○	○	essential

Clear selection

Does your paper address subitem 5-iv?

Copy and paste relevant sections from the manuscript (include quotes in quotation marks "like this" to indicate direct quotes from your manuscript), or elaborate on this item by providing additional information not in the ms, or briefly explain why the item is not applicable/relevant for your study

"Due to the COVID-19 pandemic, post-test one (completed in April-May, 2020, three months after intervention and six months after baseline) and post-test two (completed in October-November, 2020, nine months after intervention and 12 months after baseline) were self-administered remotely via the web to ensure completion, regardless of students' location of residence. Some internet disruption was apparent, as the mean number of survey attempts was greater than one in both groups and post-test rounds. Still, agreement across survey attempts between outcome-related responses exceeded 95%."

5-v) Ensure replicability by publishing the source code, and/or providing screenshots/screen-capture video, and/or providing flowcharts of the algorithms used

Ensure replicability by publishing the source code, and/or providing screenshots/screen-capture video, and/or providing flowcharts of the algorithms used. Replicability (i.e., other researchers should in principle be able to replicate the study) is a hallmark of scientific reporting.

	1	2	3	4	5	
subitem not at all important	○	○	○	○	○	essential

Does your paper address subitem 5-v?

Copy and paste relevant sections from the manuscript (include quotes in quotation marks "like this" to indicate direct quotes from your manuscript), or elaborate on this item by providing additional information not in the ms, or briefly explain why the item is not applicable/relevant for your study

We provide screenshots in the supplemental material

5-vi) Digital preservation

Digital preservation: Provide the URL of the application, but as the intervention is likely to change or disappear over the course of the years; also make sure the intervention is archived (Internet Archive, webcitation.org, and/or publishing the source code or screenshots/videos alongside the article). As pages behind login screens cannot be archived, consider creating demo pages which are accessible without login.

	1	2	3	4	5	
subitem not at all important	○	○	◉	○	○	essential

Clear selection

Does your paper address subitem 5-vi?

Copy and paste relevant sections from the manuscript (include quotes in quotation marks "like this" to indicate direct quotes from your manuscript), or elaborate on this item by providing additional information not in the ms, or briefly explain why the item is not applicable/relevant for your study

We provide screenshots in the supplemental material

5-vii) Access

Access: Describe how participants accessed the application, in what setting/context, if they had to pay (or were paid) or not, whether they had to be a member of specific group. If known, describe how participants obtained “access to the platform and Internet” [1]. To ensure access for editors/reviewers/readers, consider to provide a “backdoor” login account or demo mode for reviewers/readers to explore the application (also important for archiving purposes, see vi).

	1	2	3	4	5	
subitem not at all important	○	○	○	◉	○	essential

Clear selection

Does your paper address subitem 5-vii? *

Copy and paste relevant sections from the manuscript (include quotes in quotation marks "like this" to indicate direct quotes from your manuscript), or elaborate on this item by providing additional information not in the ms, or briefly explain why the item is not applicable/relevant for your study

"The program was formatted for delivery to computers and smartphones. Participants were provided unique login information to access the platform and could work at their own pace, could not skip segments, and were encouraged by email and text messages to complete all modules in 12 weeks."

5-viii) Mode of delivery, features/functionalities/components of the intervention and comparator, and the theoretical framework

Describe mode of delivery, features/functionalities/components of the intervention and comparator, and the theoretical framework [6] used to design them (instructional strategy [1], behaviour change techniques, persuasive features, etc., see e.g., [7, 8] for terminology). This includes an in-depth description of the content (including where it is coming from and who developed it) [1]," whether [and how] it is tailored to individual circumstances and allows users to track their progress and receive feedback" [6]. This also includes a description of communication delivery channels and – if computer-mediated communication is a component – whether communication was synchronous or asynchronous [6]. It also includes information on presentation strategies [1], including page design principles, average amount of text on pages, presence of hyperlinks to other resources, etc. [1].

	1	2	3	4	5	
subitem not at all important	○	○	○	○	◉	essential

Clear selection

Does your paper address subitem 5-viii? *

Copy and paste relevant sections from the manuscript (include quotes in quotation marks "like this" to indicate direct quotes from your manuscript), or elaborate on this item by providing additional information not in the ms, or briefly explain why the item is not applicable/relevant for your study

"The final GlobalConsent program included six, 30-minute modules, each ranging in the number of segments and types of activities, with diverse actors and language suitable for male students in Vietnam (Supplemental File 1). Modules—grounded in social cognitive theory, social norms theory and a bystander framework [37]—covered six topics to address hypothesized knowledge, normative/attitudinal, and affective mediators of the program and behavioral outcomes. These topics were consent for sex, rape-myth beliefs and norms about gender roles, effective communication, alcohol and rape, victim empathy, and bystander intervention. Each module included didactic activities, interactivity, and episodes of an adapted serial drama that modeled positive behaviors with male peers and female sex partners. Participants were provided unique login information to access the platform and could work at their own pace, could not skip segments, and were encouraged by email and text messages to complete all modules in 12 weeks."

5-ix) Describe use parameters

Describe use parameters (e.g., intended “doses” and optimal timing for use). Clarify what instructions or recommendations were given to the user, e.g., regarding timing, frequency, heaviness of use, if any, or was the intervention used ad libitum.

	1	2	3	4	5	
subitem not at all important	○	○	○	○	○	essential

Does your paper address subitem 5-ix?

Copy and paste relevant sections from the manuscript (include quotes in quotation marks "like this" to indicate direct quotes from your manuscript), or elaborate on this item by providing additional information not in the ms, or briefly explain why the item is not applicable/relevant for your study

"The final GlobalConsent program included six, 30-minute modules...Participants were provided unique login information to access the platform and could work at their own pace, could not skip segments, and were encouraged by email and text messages to complete all modules in 12 weeks."

5-x) Clarify the level of human involvement

Clarify the level of human involvement (care providers or health professionals, also technical assistance) in the e-intervention or as co-intervention (detail number and expertise of professionals involved, if any, as well as “type of assistance offered, the timing and frequency of the support, how it is initiated, and the medium by which the assistance is delivered”. It may be necessary to distinguish between the level of human involvement required for the trial, and the level of human involvement required for a routine application outside of a RCT setting (discuss under item 21 – generalizability).

	1	2	3	4	5	
subitem not at all important	○	●	○	○	○	essential

Clear selection

Does your paper address subitem 5-x?

Copy and paste relevant sections from the manuscript (include quotes in quotation marks "like this" to indicate direct quotes from your manuscript), or elaborate on this item by providing additional information not in the ms, or briefly explain why the item is not applicable/relevant for your study

This is not mentioned in the manuscript, because participants were not provided with assistance in accessing the intervention.

5-xi) Report any prompts/reminders used

Report any prompts/reminders used: Clarify if there were prompts (letters, emails, phone calls, SMS) to use the application, what triggered them, frequency etc. It may be necessary to distinguish between the level of prompts/reminders required for the trial, and the level of prompts/reminders for a routine application outside of a RCT setting (discuss under item 21 – generalizability).

	1	2	3	4	5	
subitem not at all important	○	○	●	○	○	essential

Clear selection

Does your paper address subitem 5-xi? *

Copy and paste relevant sections from the manuscript (include quotes in quotation marks "like this" to indicate direct quotes from your manuscript), or elaborate on this item by providing additional information not in the ms, or briefly explain why the item is not applicable/relevant for your study

"Participants were provided unique login information to access the platform and could work at their own pace, could not skip segments, and were encouraged by email and text messages to complete all modules in 12 weeks."

5-xii) Describe any co-interventions (incl. training/support)

Describe any co-interventions (incl. training/support): Clearly state any interventions that are provided in addition to the targeted eHealth intervention, as ehealth intervention may not be designed as stand-alone intervention. This includes training sessions and support [1]. It may be necessary to distinguish between the level of training required for the trial, and the level of training for a routine application outside of a RCT setting (discuss under item 21 – generalizability.

	1	2	3	4	5	
subitem not at all important	(●)	( )	( )	( )	( )	essential

Clear selection

Does your paper address subitem 5-xii? *

Copy and paste relevant sections from the manuscript (include quotes in quotation marks "like this" to indicate direct quotes from your manuscript), or elaborate on this item by providing additional information not in the ms, or briefly explain why the item is not applicable/relevant for your study

No cointerventions are described in the manuscript as none were performed

6a) Completely defined pre-specified primary and secondary outcome measures, including how and when they were assessed

Does your paper address CONSORT subitem 6a? *

Copy and paste relevant sections from the manuscript (include quotes in quotation marks "like this" to indicate direct quotes from your manuscript), or elaborate on this item by providing additional information not in the ms, or briefly explain why the item is not applicable/relevant for your study

"Primary outcomes. The primary outcomes for this analysis were sexually violent behavior, measured using the Sexual Experiences Survey [44], and prosocial bystander behavior, measured using four items from an adapted Bystander Behavior Scale [45] (Table 1). The Sexual Experiences Survey asks about the perpetration of seven acts of contact sexual violence, ranging from unwanted touching to forced penetration, using any of seven physical or non-physical tactics, such as holding someone down or threatening to end the relationship (35 items total). The Sexual Experiences Survey also asks about the perpetration of 10 acts of non-contact sexual violence, such as masturbating in front of someone when they did not agree. We captured sexually violent behavior based on the reported frequency (never, once, twice, 3 or more times) of all 45 items in the prior six months. We captured prosocial bystander behavior based on the reported frequency (never, 1 time, 2 or more times) in the prior 12 months of four acts, such as “I have talked with someone about sexual or dating violence as an issue for our university.” The frequency of missingness was 0.13%-0.68% across all items, and these responses were recoded as 0, “not reported.” We created kernel density plots of the summative scores for each primary outcome (Supplemental File 2). Based on their distributions, we captured three measurement scales for each behavioral outcome to assess whether the GlobalConsent program had linear or threshold effects. These measurement scales were: any reported act (1 or more vs 0 or none reported), many reported acts (3 or more vs 0-2 or none reported), and number of reported acts (Table 1)."

6a-i) Online questionnaires: describe if they were validated for online use and apply CHERRIES items to describe how the questionnaires were designed/deployed

If outcomes were obtained through online questionnaires, describe if they were validated for online use and apply CHERRIES items to describe how the questionnaires were designed/deployed [9].

	1	2	3	4	5	
subitem not at all important	○	○	○	○	◉	essential

Clear selection

Does your paper address subitem 6a-i?

Copy and paste relevant sections from manuscript text

"Eligible, consenting men who completed an in-person, computer-assisted self-administered baseline survey at their home university in September 2019 (n=793)...Due to the COVID-19 pandemic, post-test one (completed in April-May, 2020, three months after intervention and six months after baseline) and post-test two (completed in October-November, 2020, nine months after intervention and 12 months after baseline) were self-administered remotely via the web to ensure completion, regardless of students' location of residence."

We do not include additional detail regarding data collection procedures, as these have been covered in the protocol paper, which can be accessed at https://link.springer.com/article/10.1186/s12889-020-09454-2. Relevant sections from this paper include:

"Data on outcomes, mediators, and controls were assessed at baseline (wave 1; in September 2019) and at six months post-baseline (Wave 2; 12 weeks after finishing the last program module), and will be assessed at 12 months post-baseline (Wave 3) (Supplementary File 2. Quantitative Assessment Forms). The outcomes, exposure, mediators, and control variables are summarized in Table 5, along with the wave in which each measure has been (will be) included. We have considered confounders and modifiers, such as family background, exposure to violence in childhood, and exposure to online and other forms of explicit sexual content (Table 5). Assessment forms, scales, and questions were adapted, as needed, from the original RealConsent assessment forms and others in the field to be appropriate for the local context and to align with the adapted learning modules [66]...Each participant has been budgeted to receive graduated payments for completing the baseline assessment, six-month follow-up assessment, and 12-month follow-up assessment ($5 and $8 paid for Baseline and Wave 2 data collection, $10 to be paid after Wave 3 data collection; total $25) as well as $2.5 for each learning module ($15 for all six modules). University staff have been paying the students directly when they finish each survey and when they complete the learning program.
Data systems were designed to handle the secure collection, processing, storage, and analysis of project data. Data-collection modules for each wave were built into a REDCap project and delivered at baseline to participants via tablets using the REDCap mobile app, which can collect data offline and store and transmit it securely to a server when internet connectivity is available. The web application has been transmitting data entered by each participant through an encrypted network connection to a centralized database running on a secure network and infrastructure, ensuring secure electronic data movement. Each participant was assigned a unique random number identifying him across study waves and REDCap projects. All identifiable data (names, contact info, etc.) and randomized treatment/control arm assignment has been stored with the unique ID in a separate REDCap project not accessible to the Emory study team. The Emory study team will receive randomization data only after completion of the main study analyses. CCIHP and Emory staff have created and are running applications for more refined range/consistency checks in the centralized database. Systematic data collection errors for baseline and Wave 2 data collection will be identified, resolved, and documented using the logging application in REDCap. For analyses done with external statistical software, de-identified data will be

extracted and held on HIPAA compliant secure networks and computing workstations."
"All data have been and will continue to be collected in accordance with established ethical guidelines for research on gender-based violence, including review and approval by the Institutional Review Board of Emory University (IRB00099860) and the Hanoi University of Public Health (017–384/DD-YTCC). Oral consent forms for adults were developed for each type of interview (e.g., SSI, FGD, survey). The oral consent form covered the following topics: introduction of interviewer and institution s/he represents; purpose of the study: study procedures; anticipated risks or discomfort; benefits; confidentiality; right to refuse or withdraw from the study; contact details of the study coordinators for any questions or concerns; contact details for Emory and CCIHP for any complaints about treatment during the study."

6a-ii) Describe whether and how “use” (including intensity of use/dosage) was defined/measured/monitored

Describe whether and how “use” (including intensity of use/dosage) was defined/measured/monitored (logins, logfile analysis, etc.). Use/adoption metrics are important process outcomes that should be reported in any ehealth trial.

	1	2	3	4	5	
subitem not at all important	○	○	○	◉	○	essential

Clear selection

Does your paper address subitem 6a-ii?

Copy and paste relevant sections from manuscript text

"After baseline, high percentages of men in both the GlobalConsent and AHEAD groups, respectively, logged into the program (94.9%, 97.7%), completed at least one module (91.4%, 94.4%), and completed all modules (88.9%, 91.2%)." From protocol paper: "GlobalConsent and AHEAD were delivered via a secure Web portal to smartphones, so human delivery errors were minimized. We were able to monitor login/logout times, frequency of logging in/out, and total time studying each module. One week after launching the learning programs, any student who had not logged in received a reminder via email and SMS to his smartphone. Students who had not completed the modules as planned (for this study, one module per week) received encouraging messages via email and smartphone. After two reminders via email and SMS, the study team at CCIHP contacted students who still had not advanced in the program by calling directly or contacting indirectly via a general Facebook group with non-named members and connections visible only to the Facebook account administrator. This behavioral data will be used to assess program completion, dose of exposure (number of modules completed for those who did not complete the program), and eventually, any refinements needed for the web application."

6a-iii) Describe whether, how, and when qualitative feedback from participants was obtained

Describe whether, how, and when qualitative feedback from participants was obtained (e.g., through emails, feedback forms, interviews, focus groups).

	1	2	3	4	5	
subitem not at all important	○	○	◉	○	○	essential

Clear selection

Does your paper address subitem 6a-iii?

Copy and paste relevant sections from manuscript text

Qualitative feedback was not obtained post-intervention. However, qualitative feedback was obtained prior to the intervention during formative research. "The adapted GlobalConsent program took into account qualitative findings [38, 40] and feedback from focus group discussions with male students and university stakeholders on the RealConsent program and the adapted GlobalConsent storyboards before production (Supplemental File 1)."

6b) Any changes to trial outcomes after the trial commenced, with reasons

Does your paper address CONSORT subitem 6b? *

Copy and paste relevant sections from the manuscript (include quotes in quotation marks "like this" to indicate direct quotes from your manuscript), or elaborate on this item by providing additional information not in the ms, or briefly explain why the item is not applicable/relevant for your study

There were no changes to trial outcomes after the trial commenced.

7a) How sample size was determined

NPT: When applicable, details of whether and how the clustering by care provides or centers was addressed

7a-i) Describe whether and how expected attrition was taken into account when calculating the sample size

Describe whether and how expected attrition was taken into account when calculating the sample size.

	1	2	3	4	5	
subitem not at all important	○	○	○	●	○	essential

Clear selection

Does your paper address subitem 7a-i?

Copy and paste relevant sections from manuscript title (include quotes in quotation marks "like this" to indicate direct quotes from your manuscript), or elaborate on this item by providing additional information not in the ms, or briefly explain why the item is not applicable/relevant for your study

Power calculations are described in detail in the protocol paper:

From the protocol paper: "Men 18–24 years and self-identifying as heterosexual or bisexual and enrolled at HMU and TLU as freshmen on 9/1/2019 were eligible. According to recent enrollment figures, about 890 men matriculate yearly at HMU and TLU. Assuming a cooperation rate of 90% based on prior studies of this team in Vietnam [12], this pool was considered sufficient to recruit a sample of 800 freshman men for participation in this study.

We computed the required sample sizes to detect our hypothesized effects with a power of .80. Figure 3 depicts how the effect of GlobalConsent on one of the outcome variables, bystander behavior, is transmitted through one of the mediator variables, Rape Myth Acceptance [101, 102]. In the model, a represents the causal pathway between the GlobalConsent treatment and the Bystander Behavior Scale, and the product ab captures the indirect effect of GlobalConsent on Bystander Behavior through Rape Myth Acceptance. We used a Monte Carlo approach [103] to calculate the required sample sizes assuming 1) an attrition rate of 10%; 2) Cohen's small-to-large-effect-categorization [104] regarding the proportion of the variance accounted for, R2, by the model (.02, .13 and .26) using specific values for a and b [105,106,107]; and 3) a desired power level of .80. The computer program, Mplus [108], was used to complete the computations. Results of the simulation suggested that our final sample size of 800 had adequate power to detect small, medium and large a and b, and ab for the mediation model (Table 2)."

7b) When applicable, explanation of any interim analyses and stopping guidelines

Does your paper address CONSORT subitem 7b? *

Copy and paste relevant sections from the manuscript (include quotes in quotation marks "like this" to indicate direct quotes from your manuscript), or elaborate on this item by providing additional information not in the ms, or briefly explain why the item is not applicable/relevant for your study

This item is not applicable as there were no interim analyses or stopping guidelines.

8a) Method used to generate the random allocation sequence

NPT: When applicable, how care providers were allocated to each trial group

Does your paper address CONSORT subitem 8a? *

Copy and paste relevant sections from the manuscript (include quotes in quotation marks "like this" to indicate direct quotes from your manuscript), or elaborate on this item by providing additional information not in the ms, or briefly explain why the item is not applicable/relevant for your study

Not applicable: There were no care providers in this study.

8b) Type of randomisation; details of any restriction (such as blocking and block size)

Does your paper address CONSORT subitem 8b? *

Copy and paste relevant sections from the manuscript (include quotes in quotation marks "like this" to indicate direct quotes from your manuscript), or elaborate on this item by providing additional information not in the ms, or briefly explain why the item is not applicable/relevant for your study

"Eligible, consenting men who completed an in-person, computer-assisted self-administered baseline survey at their home university in September 2019 (n=793) each were assigned a random number generated in excel, sorted in ascending order. The first 397 men were assigned to the AHEAD attention-control group, and the remaining men were assigned to the GlobalConsent group."

9) Mechanism used to implement the random allocation sequence (such as sequentially numbered containers), describing any steps taken to conceal the sequence until interventions were assigned

Does your paper address CONSORT subitem 9? *

Copy and paste relevant sections from the manuscript (include quotes in quotation marks "like this" to indicate direct quotes from your manuscript), or elaborate on this item by providing additional information not in the ms, or briefly explain why the item is not applicable/relevant for your study

"Eligible, consenting men who completed an in-person, computer-assisted self-administered baseline survey at their home university in September 2019 (n=793) each were assigned a random number generated in excel, sorted in ascending order. The first 397 men were assigned to the AHEAD attention-control group, and the remaining men were assigned to the GlobalConsent group."

10) Who generated the random allocation sequence, who enrolled participants, and who assigned participants to interventions

Does your paper address CONSORT subitem 10? *

Copy and paste relevant sections from the manuscript (include quotes in quotation marks "like this" to indicate direct quotes from your manuscript), or elaborate on this item by providing additional information not in the ms, or briefly explain why the item is not applicable/relevant for your study

"Eligible, consenting men who completed an in-person, computer-assisted self-administered baseline survey at their home university in September 2019 (n=793) each were assigned a random number generated in excel, sorted in ascending order. The first 397 men were assigned to the AHEAD attention-control group, and the remaining men were assigned to the GlobalConsent group."

11a) If done, who was blinded after assignment to interventions (for example, participants, care providers, those assessing outcomes) and how

NPT: Whether or not administering co-interventions were blinded to group assignment

11a-i) Specify who was blinded, and who wasn’t

Specify who was blinded, and who wasn’t. Usually, in web-based trials it is not possible to blind the participants [1, 3] (this should be clearly acknowledged), but it may be possible to blind outcome assessors, those doing data analysis or those administering co-interventions (if any).

	1	2	3	4	5	
subitem not at all important	○	○	○	◉	○	essential

Clear selection

Does your paper address subitem 11a-i? *

Copy and paste relevant sections from the manuscript (include quotes in quotation marks "like this" to indicate direct quotes from your manuscript), or elaborate on this item by providing additional information not in the ms, or briefly explain why the item is not applicable/relevant for your study

"Participants were blinded to these assignments because of our use of an attention-control condition, and the analysis team was blinded to these assignments throughout the analysis."

11a-ii) Discuss e.g., whether participants knew which intervention was the “intervention of interest” and which one was the “comparator”

Informed consent procedures (4a-ii) can create biases and certain expectations - discuss e.g., whether participants knew which intervention was the “intervention of interest” and which one was the “comparator”.

	1	2	3	4	5	
subitem not at all important	○	○	○	◉	○	essential

Clear selection

Does your paper address subitem 11a-ii?

Copy and paste relevant sections from the manuscript (include quotes in quotation marks "like this" to indicate direct quotes from your manuscript), or elaborate on this item by providing additional information not in the ms, or briefly explain why the item is not applicable/relevant for your study

"Participants were blinded to these assignments because of our use of an attention-control condition, and the analysis team was blinded to these assignments throughout the analysis."

11b) If relevant, description of the similarity of interventions

(this item is usually not relevant for ehealth trials as it refers to similarity of a placebo or sham intervention to a active medication/intervention)

Does your paper address CONSORT subitem 11b? *

Copy and paste relevant sections from the manuscript (include quotes in quotation marks "like this" to indicate direct quotes from your manuscript), or elaborate on this item by providing additional information not in the ms, or briefly explain why the item is not applicable/relevant for your study

"AHEAD Attention-Control Program
The team developed Adolescent Health Education (AHEAD), the health-education, attention-control program, from open-access content suitable for young people in Vietnam [37]. To approximate the format, intensity, and duration of GlobalConsent, AHEAD was developed as a web-delivered, multimedia program audio-narrated in Vietnamese with six 35–45 minute learning modules on brain development, nutrition, physical activity, substance use, sleep, and agency. Like GlobalConsent, participants could work at their own pace, could not skip segments, and were encouraged by email and text messages to complete all modules in 12 weeks."

12a) Statistical methods used to compare groups for primary and secondary outcomes

NPT: When applicable, details of whether and how the clustering by care providers or centers was addressed

Does your paper address CONSORT subitem 12a? *

Copy and paste relevant sections from the manuscript (include quotes in quotation marks "like this" to indicate direct quotes from your manuscript), or elaborate on this item by providing additional information not in the ms, or briefly explain why the item is not applicable/relevant for your study

"First, we conducted descriptive analyses of the sample, overall and by study arm to assess qualitatively balance across groups. Second, we assessed levels of sexually violent behavior and prosocial intervening behavior at each study wave by study arm. Finally, we conducted difference-in-difference (DID) modeling to estimate the odds ratios (OR) and incidence rate ratios (IRR) and 95% confidence intervals (CI) for sexually violent behavior and prosocial bystander behavior at endline (posttest 1 and 2 combined) versus baseline. For binary outcomes, we performed logistic regression with treatment arm, time, and the treatment-by-time interaction (the DID variable) as predictors. For count outcomes, we performed negative binomial regression, which accounts for over-dispersion in the outcome. To probe significant treatment-by-time interaction or DID effects, we computed and compared the OR and/or IRR of the occurrence of the behavior separately for each group."

12a-i) Imputation techniques to deal with attrition / missing values

Imputation techniques to deal with attrition / missing values: Not all participants will use the intervention/comparator as intended and attrition is typically high in ehealth trials. Specify how participants who did not use the application or dropped out from the trial were treated in the statistical analysis (a complete case analysis is strongly discouraged, and simple imputation techniques such as LOCF may also be problematic [4]).

	1	2	3	4	5	
subitem not at all important	○	○	○	●	○	essential

Clear selection

Does your paper address subitem 12a-i? *

Copy and paste relevant sections from the manuscript (include quotes in quotation marks "like this" to indicate direct quotes from your manuscript), or elaborate on this item by providing additional information not in the ms, or briefly explain why the item is not applicable/relevant for your study

Imputation techniques were not used because of low attrition and high completeness of data. Loss to follow up overall was less than 7% of the baseline sample.

"We captured sexually violent behavior based on the reported frequency (never, once, twice, 3 or more times) of all 45 items in the prior six months. We captured prosocial bystander behavior based on the reported frequency (never, 1 time, 2 or more times) in the prior 12 months of four acts, such as “I have talked with someone about sexual or dating violence as an issue for our university.” The frequency of missingness was 0.13%-0.68% across all items, and these responses were recoded as 0, “not reported.”

12b) Methods for additional analyses, such as subgroup analyses and adjusted analyses

Does your paper address CONSORT subitem 12b? *

Copy and paste relevant sections from the manuscript (include quotes in quotation marks "like this" to indicate direct quotes from your manuscript), or elaborate on this item by providing additional information not in the ms, or briefly explain why the item is not applicable/relevant for your study

Not applicable, no secondary analyses have been performed.

X26) REB/IRB Approval and Ethical Considerations [recommended as subheading under "Methods"] (not a CONSORT item)

X26-i) Comment on ethics committee approval

	1	2	3	4	5	
subitem not at all important	○	○	○	◉	○	essential

Clear selection

Does your paper address subitem X26-i?

Copy and paste relevant sections from the manuscript (include quotes in quotation marks "like this" to indicate direct quotes from your manuscript), or elaborate on this item by providing additional information not in the ms, or briefly explain why the item is not applicable/relevant for your study

"Data were collected following established ethical guidelines for research on gender-based violence [37]. The Institutional Review Boards of Emory University (IRB00099860) and the Hanoi University of Public Health (017–384/DD-YTCC) reviewed and approved the study protocol, including online consent forms used in the study."

x26-ii) Outline informed consent procedures

Outline informed consent procedures e.g., if consent was obtained offline or online (how? Checkbox, etc.?), and what information was provided (see 4a-ii). See [6] for some items to be included in informed consent documents.

	1	2	3	4	5	
subitem not at all important	○	○	○	◉	○	essential

Clear selection

Does your paper address subitem X26-ii?

Copy and paste relevant sections from the manuscript (include quotes in quotation marks "like this" to indicate direct quotes from your manuscript), or elaborate on this item by providing additional information not in the ms, or briefly explain why the item is not applicable/relevant for your study

"Data were collected following established ethical guidelines for research on gender-based violence [37]. The Institutional Review Boards of Emory University (IRB00099860) and the Hanoi University of Public Health (017–384/DD-YTCC) reviewed and approved the study protocol, including online consent forms used in the study."

From the protocol paper: "Students who agreed to participate were invited to different sessions organized in a meeting hall or classes where they could complete a tablet-assisted informed consent process and self-interview."

X26-iii) Safety and security procedures

Safety and security procedures, incl. privacy considerations, and any steps taken to reduce the likelihood or detection of harm (e.g., education and training, availability of a hotline)

	1	2	3	4	5	
subitem not at all important	○	○	○	◉	○	essential

Clear selection

Does your paper address subitem X26-iii?

Copy and paste relevant sections from the manuscript (include quotes in quotation marks "like this" to indicate direct quotes from your manuscript), or elaborate on this item by providing additional information not in the ms, or briefly explain why the item is not applicable/relevant for your study

While not explicitly addressed in the current manuscript, this information is provided in the protocol paper: "The web application has been transmitting data entered by each participant through an encrypted network connection to a centralized database running on a secure network and infrastructure, ensuring secure electronic data movement. Each participant was assigned a unique random number identifying him across study waves and REDCap projects. All identifiable data (names, contact info, etc.) and randomized treatment/control arm assignment has been stored with the unique ID in a separate REDCap project not accessible to the Emory study team. The Emory study team will receive randomization data only after completion of the main study analyses. CCIHP and Emory staff have created and are running applications for more refined range/consistency checks in the centralized database. Systematic data collection errors for baseline and Wave 2 data collection will be identified, resolved, and documented using the logging application in REDCap. For analyses done with external statistical software, de-identified data will be extracted and held on HIPAA compliant secure networks and computing workstations."

RESULTS

13a) For each group, the numbers of participants who were randomly assigned, received intended treatment, and were analysed for the primary outcome

NPT: The number of care providers or centers performing the intervention in each group and the number of patients treated by each care provider in each center

Does your paper address CONSORT subitem 13a? *

Copy and paste relevant sections from the manuscript (include quotes in quotation marks "like this" to indicate direct quotes from your manuscript), or elaborate on this item by providing additional information not in the ms, or briefly explain why the item is not applicable/relevant for your study

"Eligible, consenting men who completed an in-person, computer-assisted self-administered baseline survey at their home university in September 2019 (n=793) each were assigned a random number generated in excel, sorted in ascending order. The first 397 men were assigned to the AHEAD attention-control group, and the remaining men were assigned to the GlobalConsent group...In the GlobalConsent group, 374 and 364 of 396 students completed post-test one and two, respectively. In the AHEAD group, 377 and 375 of 397 students completed post-test one and two, respectively."

13b) For each group, losses and exclusions after randomisation, together with reasons

Does your paper address CONSORT subitem 13b? (NOTE: Preferably, this is shown in a CONSORT flow diagram) *

Copy and paste relevant sections from the manuscript (include quotes in quotation marks "like this" to indicate direct quotes from your manuscript), or elaborate on this item by providing additional information not in the ms, or briefly explain why the item is not applicable/relevant for your study

"Six assigned participants (four GlobalConsent, two AHEAD) declined to login to their assigned learning program." Figure 1 shows the CONSORT flow diagram in greater detail at each stage.

13b-i) Attrition diagram

Strongly recommended: An attrition diagram (e.g., proportion of participants still logging in or using the intervention/comparator in each group plotted over time, similar to a survival curve) or other figures or tables demonstrating usage/dose/engagement.

	1	2	3	4	5	
subitem not at all important	○	○	○	○	◉	essential

Clear selection

Does your paper address subitem 13b-i?

Copy and paste relevant sections from the manuscript or cite the figure number if applicable (include quotes in quotation marks "like this" to indicate direct quotes from your manuscript), or elaborate on this item by providing additional information not in the ms, or briefly explain why the item is not applicable/relevant for your study

This is Figure 1 in the manuscript.

14a) Dates defining the periods of recruitment and follow-up

Does your paper address CONSORT subitem 14a? *

Copy and paste relevant sections from the manuscript (include quotes in quotation marks "like this" to indicate direct quotes from your manuscript), or elaborate on this item by providing additional information not in the ms, or briefly explain why the item is not applicable/relevant for your study

"Eligible, consenting men who completed an in-person, computer-assisted self-administered baseline survey at their home university in September 2019...Participants were able to access their assigned program from November 2019 to January 2020....Due to the COVID-19 pandemic, post-test one (completed in April-May, 2020, three months after intervention and six months after baseline) and post-test two (completed in October-November, 2020, nine months after intervention and 12 months after baseline) were self-administered remotely via the web to ensure completion."

14a-i) Indicate if critical “secular events” fell into the study period

Indicate if critical “secular events” fell into the study period, e.g., significant changes in Internet resources available or “changes in computer hardware or Internet delivery resources”

	1	2	3	4	5	
subitem not at all important	○	○	●	○	○	essential

Clear selection

Does your paper address subitem 14a-i?

Copy and paste relevant sections from the manuscript (include quotes in quotation marks "like this" to indicate direct quotes from your manuscript), or elaborate on this item by providing additional information not in the ms, or briefly explain why the item is not applicable/relevant for your study

"Due to the COVID-19 pandemic, post-test one (completed in April-May, 2020, three months after intervention and six months after baseline) and post-test two (completed in October-November, 2020, nine months after intervention and 12 months after baseline) were self-administered remotely via the web to ensure completion, regardless of students' location of residence. Some internet disruption was apparent, as the mean number of survey attempts was greater than one in both groups and post-test rounds. Still, agreement across survey attempts between outcome-related responses exceeded 95%."

14b) Why the trial ended or was stopped (early)

Does your paper address CONSORT subitem 14b? *

Copy and paste relevant sections from the manuscript (include quotes in quotation marks "like this" to indicate direct quotes from your manuscript), or elaborate on this item by providing additional information not in the ms, or briefly explain why the item is not applicable/relevant for your study

"post-test one (completed in April-May, 2020, three months after intervention and six months after baseline) and post-test two for trial endline (completed in October-November, 2020, nine months after intervention and 12 months after baseline)". There is no further detail because the trial was not stopped early.

15) A table showing baseline demographic and clinical characteristics for each group

NPT: When applicable, a description of care providers (case volume, qualification, expertise, etc.) and centers (volume) in each group

Does your paper address CONSORT subitem 15? *

Copy and paste relevant sections from the manuscript (include quotes in quotation marks "like this" to indicate direct quotes from your manuscript), or elaborate on this item by providing additional information not in the ms, or briefly explain why the item is not applicable/relevant for your study

This is Table 2 in the manuscript.

15-i) Report demographics associated with digital divide issues

In ehealth trials it is particularly important to report demographics associated with digital divide issues, such as age, education, gender, social-economic status, computer/Internet/ehealth literacy of the participants, if known.

	1	2	3	4	5	
subitem not at all important	○	○	○	○	○	essential

Does your paper address subitem 15-i? *

Copy and paste relevant sections from the manuscript (include quotes in quotation marks "like this" to indicate direct quotes from your manuscript), or elaborate on this item by providing additional information not in the ms, or briefly explain why the item is not applicable/relevant for your study

Table 2 shows participant ages and ethnicities. Education, gender, and computer literacy are not shown since eligible participants are male first-year university students.

16) For each group, number of participants (denominator) included in each analysis and whether the analysis was by original assigned groups

16-i) Report multiple “denominators” and provide definitions

Report multiple “denominators” and provide definitions: Report N’s (and effect sizes) “across a range of study participation [and use] thresholds” [1], e.g., N exposed, N consented, N used more than x times, N used more than y weeks, N participants “used” the intervention/comparator at specific pre-defined time points of interest (in absolute and relative numbers per group). Always clearly define “use” of the intervention.

	1	2	3	4	5	
subitem not at all important	○	○	○	◉	○	essential

Clear selection

Does your paper address subitem 16-i? *

Copy and paste relevant sections from the manuscript (include quotes in quotation marks "like this" to indicate direct quotes from your manuscript), or elaborate on this item by providing additional information not in the ms, or briefly explain why the item is not applicable/relevant for your study

"Reported rates of any sexually violent behavior were 31.06%, 21.39%, and 31.87% across survey waves in the GlobalConsent group, and were 25.94%, 26.26%, and 31.47% across waves in the AHEAD group (Table 3). Rates of ‘high’ sexually violent behavior (more than 2 acts) were 20.96%, 13.64%, and 24.73% across waves in the GlobalConsent group, and were 17.13%, 18.03% and 23.20% across waves in the AHEAD group. The mean count of sexually violent acts was 3.15, 2.24, and 3.07 across waves in the GlobalConsent group and 2.41, 3.55, and 3.24 across waves in the AHEAD group...Reported rates of any prosocial bystander behavior were 65.15% and 55.77% across survey waves in the GlobalConsent group and were 64.99% and 45.33% across waves in the AHEAD group (Table 3). Rates of ‘high’ prosocial bystander behavior (more than two acts) were 41.16% and 37.91% across waves in the GlobalConsent group and were 35.52% and 28.80% across waves in the AHEAD group. The mean counts of prosocial bystander acts were 2.39 and 2.33 across waves in the GlobalConsent group and were 2.20 and 1.69 across waves in the AHEAD group."
Denominators are found in Table 3.

16-ii) Primary analysis should be intent-to-treat

Primary analysis should be intent-to-treat, secondary analyses could include comparing only “users”, with the appropriate caveats that this is no longer a randomized sample (see 18-i).

	1	2	3	4	5	
subitem not at all important	○	○	○	○	◉	essential

Clear selection

Does your paper address subitem 16-ii?

Copy and paste relevant sections from the manuscript (include quotes in quotation marks "like this" to indicate direct quotes from your manuscript), or elaborate on this item by providing additional information not in the ms, or briefly explain why the item is not applicable/relevant for your study

"we performed logistic regression with assigned treatment arm, time, and the treatment-by-time interaction"

17a) For each primary and secondary outcome, results for each group, and the estimated effect size and its precision (such as 95% confidence interval)

Does your paper address CONSORT subitem 17a? *

Copy and paste relevant sections from the manuscript (include quotes in quotation marks "like this" to indicate direct quotes from your manuscript), or elaborate on this item by providing additional information not in the ms, or briefly explain why the item is not applicable/relevant for your study

"Significant interaction effects in the difference-in-difference models suggested favorable impacts of GlobalConsent relative to AHEAD on sexually violent behavior (more than two acts) (OR = 0.47, p = 0.001, 95% CI (0.31, 0.72) and prosocial bystander behavior (any act) (OR = 1.51, p = 0.050, 95% CI (1.00, 2.28) (Table 4)." Table 4 provides more detail.

17a-i) Presentation of process outcomes such as metrics of use and intensity of use

In addition to primary/secondary (clinical) outcomes, the presentation of process outcomes such as metrics of use and intensity of use (dose, exposure) and their operational definitions is critical. This does not only refer to metrics of attrition (13-b) (often a binary variable), but also to more continuous exposure metrics such as “average session length”. These must be accompanied by a technical description how a metric like a “session” is defined (e.g., timeout after idle time) [1] (report under item 6a).

	1	2	3	4	5	
subitem not at all important	○	○	◉	○	○	essential

Clear selection

Does your paper address subitem 17a-i?

Copy and paste relevant sections from the manuscript (include quotes in quotation marks "like this" to indicate direct quotes from your manuscript), or elaborate on this item by providing additional information not in the ms, or briefly explain why the item is not applicable/relevant for your study

"After baseline, high percentages of men in both the GlobalConsent and AHEAD groups, respectively, logged into the program (94.9%, 97.7%), completed at least one module (91.4%, 94.4%), and completed all modules (88.9%, 91.2%)."

17b) For binary outcomes, presentation of both absolute and relative effect sizes is recommended

Does your paper address CONSORT subitem 17b? *

Copy and paste relevant sections from the manuscript (include quotes in quotation marks "like this" to indicate direct quotes from your manuscript), or elaborate on this item by providing additional information not in the ms, or briefly explain why the item is not applicable/relevant for your study

"Reported rates of any sexually violent behavior were 31.06%, 21.39%, and 31.87% across survey waves in the GlobalConsent group, and were 25.94%, 26.26%, and 31.47% across waves in the AHEAD group (Table 3). Rates of ‘high’ sexually violent behavior (more than 2 acts) were 20.96%, 13.64%, and 24.73% across waves in the GlobalConsent group, and were 17.13%, 18.03% and 23.20% across waves in the AHEAD group....Significant interaction effects in the difference-in-difference models suggested favorable impacts of GlobalConsent relative to AHEAD on sexually violent behavior (more than two acts) (OR = 0.47, p = 0.001, 95% CI (0.31, 0.72) and prosocial bystander behavior (any act) (OR = 1.51, p = 0.050, 95% CI (1.00, 2.28) (Table 4). Marginally significant interaction effects in DID models for any sexually violent behavior, the count for sexually violent acts, and the count for prosocial bystander acts suggested a consistent, favorable impact of GlobalConsent relative to AHEAD across measurement scales of these two behavioral outcomes.... To probe the interaction effects further, we computed separately for the GlobalConsent and AHEAD groups the odds ratios of sexually violent behavior and prosocial bystander behavior at endline (posttest 1 and 2 combined) versus baseline. Among the GlobalConsent participants, the odds of perpetrating more than two acts of sexual violence at endline were 1.27 times the odds at baseline. For the AHEAD group, the odds of perpetrating more than two acts of sexual violence at endline were 2.69 times the odds at baseline. Among the GlobalConsent participants, the odds of any prosocial behavior at posttest were 0.67 times the odds at baseline. For the AHEAD group, the odds of any prosocial behavior at posttest were 0.45 times the odds at baseline."

18) Results of any other analyses performed, including subgroup analyses and adjusted analyses, distinguishing pre-specified from exploratory

Does your paper address CONSORT subitem 18? *

Copy and paste relevant sections from the manuscript (include quotes in quotation marks "like this" to indicate direct quotes from your manuscript), or elaborate on this item by providing additional information not in the ms, or briefly explain why the item is not applicable/relevant for your study

"To probe the interaction effects further, we computed separately for the GlobalConsent and AHEAD groups the odds ratios of sexually violent behavior and prosocial bystander behavior at endline (posttest 1 and 2 combined) versus baseline. Among the GlobalConsent participants, the odds of perpetrating more than two acts of sexual violence at endline were 1.27 times the odds at baseline. For the AHEAD group, the odds of perpetrating more than two acts of sexual violence at endline were 2.69 times the odds at baseline. Among the GlobalConsent participants, the odds of any prosocial behavior at posttest were 0.67 times the odds at baseline. For the AHEAD group, the odds of any prosocial behavior at posttest were 0.45 times the odds at baseline."

18-i) Subgroup analysis of comparing only users

A subgroup analysis of comparing only users is not uncommon in ehealth trials, but if done, it must be stressed that this is a self-selected sample and no longer an unbiased sample from a randomized trial (see 16-iii).

	1	2	3	4	5	
subitem not at all important	○	○	◉	○	○	essential

Clear selection

Does your paper address subitem 18-i?

Copy and paste relevant sections from the manuscript (include quotes in quotation marks "like this" to indicate direct quotes from your manuscript), or elaborate on this item by providing additional information not in the ms, or briefly explain why the item is not applicable/relevant for your study

We did not perform such an analysis.

19) All important harms or unintended effects in each group

(for specific guidance see CONSORT for harms)

Does your paper address CONSORT subitem 19? *

Copy and paste relevant sections from the manuscript (include quotes in quotation marks "like this" to indicate direct quotes from your manuscript), or elaborate on this item by providing additional information not in the ms, or briefly explain why the item is not applicable/relevant for your study

No harms or intended effects were observed.

19-i) Include privacy breaches, technical problems

Include privacy breaches, technical problems. This does not only include physical “harm” to participants, but also incidents such as perceived or real privacy breaches [1], technical problems, and other unexpected/unintended incidents. “Unintended effects” also includes unintended positive effects [2].

	1	2	3	4	5	
subitem not at all important	○	○	◉	○	○	essential

Clear selection

Does your paper address subitem 19-i?

Copy and paste relevant sections from the manuscript (include quotes in quotation marks "like this" to indicate direct quotes from your manuscript), or elaborate on this item by providing additional information not in the ms, or briefly explain why the item is not applicable/relevant for your study

No privacy breaches occurred.

19-ii) Include qualitative feedback from participants or observations from staff/researchers

Include qualitative feedback from participants or observations from staff/researchers, if available, on strengths and shortcomings of the application, especially if they point to unintended/unexpected effects or uses. This includes (if available) reasons for why people did or did not use the application as intended by the developers.

	1	2	3	4	5	
subitem not at all important	○	○	●	○	○	essential

Clear selection

Does your paper address subitem 19-ii?

Copy and paste relevant sections from the manuscript (include quotes in quotation marks "like this" to indicate direct quotes from your manuscript), or elaborate on this item by providing additional information not in the ms, or briefly explain why the item is not applicable/relevant for your study

We do not have data on this item.

DISCUSSION

22) Interpretation consistent with results, balancing benefits and harms, and considering other relevant evidence

NPT: In addition, take into account the choice of the comparator, lack of or partial blinding, and unequal expertise of care providers or centers in each group

22-i) Restate study questions and summarize the answers suggested by the data, starting with primary outcomes and process outcomes (use)

Restate study questions and summarize the answers suggested by the data, starting with primary outcomes and process outcomes (use).

	1	2	3	4	5	
subitem not at all important	○	○	○	◉	○	essential

Clear selection

Does your paper address subitem 22-i? *

Copy and paste relevant sections from the manuscript (include quotes in quotation marks "like this" to indicate direct quotes from your manuscript), or elaborate on this item by providing additional information not in the ms, or briefly explain why the item is not applicable/relevant for your study

"This randomized clinical trial recruited 793 university men in Vietnam to test the efficacy of GlobalConsent, a culturally tailored adaptation of an efficacious, theoretically-grounded web-based sexual violence prevention program for US college men [23]. In the present study, relative to the odds for men in the attention-control group, men in the GlobalConsent group had lower odds of sexually violent behavior at endline than at baseline. Also, compared to the odds for men in the AHEAD group, men in the GlobalConsent group had higher odds of any prosocial bystander behavior at endline than at baseline. The favorable impacts of GlobalConsent versus AHEAD were consistent across measurement scales of these two behavior outcomes. Such behavioral change is notable, as this is the first web-delivered sexual violence prevention program for male college students implemented in a middle-income setting, and most sexual violence prevention programs globally have measured changes in reported behavioral intentions rather than in reported behavior"

22-ii) Highlight unanswered new questions, suggest future research

Highlight unanswered new questions, suggest future research.

	1	2	3	4	5	
subitem not at all important	○	○	○	◉	○	essential

Clear selection

Does your paper address subitem 22-ii?

Copy and paste relevant sections from the manuscript (include quotes in quotation marks "like this" to indicate direct quotes from your manuscript), or elaborate on this item by providing additional information not in the ms, or briefly explain why the item is not applicable/relevant for your study

"Longer-term follow-up of participants would be beneficial to assess the impact of GlobalConsent on behavioral outcomes throughout men’s time at university, and beyond...A large-scale implementation study of GlobalConsent in universities across Vietnam would be a robust next step to assess effectiveness, and information about barriers and facilitators to university delivery of this intervention would be critical for moving GlobalConsent from research to practice."

20) Trial limitations, addressing sources of potential bias, imprecision, and, if relevant, multiplicity of analyses

20-i) Typical limitations in ehealth trials

Typical limitations in ehealth trials: Participants in ehealth trials are rarely blinded. Ehealth trials often look at a multiplicity of outcomes, increasing risk for a Type I error. Discuss biases due to non-use of the intervention/usability issues, biases through informed consent procedures, unexpected events.

	1	2	3	4	5	
subitem not at all important	○	○	○	●	○	essential

Clear selection

Does your paper address subitem 20-i? *

Copy and paste relevant sections from the manuscript (include quotes in quotation marks "like this" to indicate direct quotes from your manuscript), or elaborate on this item by providing additional information not in the ms, or briefly explain why the item is not applicable/relevant for your study

"First, the study sites were limited to two urban universities to accommodate program adaptation, production, and testing. The findings, therefore, are not generalizable to all university men in Vietnam...Second, behavioral outcomes were self-reported and may have been subject to social desirability bias. To mitigate this limitation, the team relied on a validated instrument to measure sexual violence and self-administration, which enhances privacy and may enhance honest disclosures. Third, we did not interview women on campus about changes in behavior, as reported by men, due to resource constraints and the ethical and logistical challenges of linking women's reports of men's behavior at the individual level. Fourth, some non-significant differences in behavior and attenuation of behavioral change at 12 months suggest a need for booster training after the three-hour program period. Fifth, the 12-month follow-up period allowed for a short-term impact assessment of GlobalConsent...Sixth, like other web-based interventions, the content of GlobalConsent may become outdated, and updating or future adaptations to GlobalConsent may incur new costs."

21) Generalisability (external validity, applicability) of the trial findings

NPT: External validity of the trial findings according to the intervention, comparators, patients, and care providers or centers involved in the trial

21-i) Generalizability to other populations

Generalizability to other populations: In particular, discuss generalizability to a general Internet population, outside of a RCT setting, and general patient population, including applicability of the study results for other organizations

	1	2	3	4	5	
subitem not at all important	○	○	○	◉	○	essential

Clear selection

Does your paper address subitem 21-i?

Copy and paste relevant sections from the manuscript (include quotes in quotation marks "like this" to indicate direct quotes from your manuscript), or elaborate on this item by providing additional information not in the ms, or briefly explain why the item is not applicable/relevant for your study

"First, the study sites were limited to two urban universities to accommodate program adaptation, production, and testing. The findings, therefore, are not generalizable to all university men in Vietnam."

21-ii) Discuss if there were elements in the RCT that would be different in a routine application setting

Discuss if there were elements in the RCT that would be different in a routine application setting (e.g., prompts/reminders, more human involvement, training sessions or other co-interventions) and what impact the omission of these elements could have on use, adoption, or outcomes if the intervention is applied outside of a RCT setting.

	1	2	3	4	5	
subitem not at all important	○	○	◉	○	○	essential

Clear selection

Does your paper address subitem 21-ii?

Copy and paste relevant sections from the manuscript (include quotes in quotation marks "like this" to indicate direct quotes from your manuscript), or elaborate on this item by providing additional information not in the ms, or briefly explain why the item is not applicable/relevant for your study

This item is not applicable.

OTHER INFORMATION

23) Registration number and name of trial registry

Does your paper address CONSORT subitem 23? *

Copy and paste relevant sections from the manuscript (include quotes in quotation marks "like this" to indicate direct quotes from your manuscript), or elaborate on this item by providing additional information not in the ms, or briefly explain why the item is not applicable/relevant for your study

"ClinicalTrials.gov Identifier NCT04147455"

24) Where the full trial protocol can be accessed, if available

Does your paper address CONSORT subitem 24? *

Cite a Multimedia Appendix, other reference, or copy and paste relevant sections from the manuscript (include quotes in quotation marks "like this" to indicate direct quotes from your manuscript), or elaborate on this item by providing additional information not in the ms, or briefly explain why the item is not applicable/relevant for your study

We have cited the protocol paper: "The study design, detailed elsewhere [37], applied a double-blind, parallel intervention-versus-attention-control group design with balanced (1:1) randomization."

25) Sources of funding and other support (such as supply of drugs), role of funders

Does your paper address CONSORT subitem 25? *

Copy and paste relevant sections from the manuscript (include quotes in quotation marks "like this" to indicate direct quotes from your manuscript), or elaborate on this item by providing additional information not in the ms, or briefly explain why the item is not applicable/relevant for your study

The funder wished to remain anonymous in publicly-available documents but a statement is located in the Acknowledgements: "This study was supported by a grant from an anonymous charitable foundation."

X27) Conflicts of Interest (not a CONSORT item)

X27-i) State the relation of the study team towards the system being evaluated

In addition to the usual declaration of interests (financial or otherwise), also state the relation of the study team towards the system being evaluated, i.e., state if the authors/evaluators are distinct from or identical with the developers/sponsors of the intervention.

	1	2	3	4	5	
subitem not at all important	○	○	○	◉	○	essential

Clear selection

Does your paper address subitem X27-i?

Copy and paste relevant sections from the manuscript (include quotes in quotation marks "like this" to indicate direct quotes from your manuscript), or elaborate on this item by providing additional information not in the ms, or briefly explain why the item is not applicable/relevant for your study

None of the authors/evaluators has a conflict of interest.

About the CONSORT EHEALTH checklist

As a result of using this checklist, did you make changes in your manuscript? *

- ○ yes, major changes
- ◉ yes, minor changes
- ○ no

What were the most important changes you made as a result of using this checklist?

Noting information in the abstract that was only in the manuscript body.

How much time did you spend on going through the checklist INCLUDING making changes in your manuscript *

4 hours.

Your answer must have a minimum of 25 characters.

As a result of using this checklist, do you think your manuscript has improved? *

- ( ) yes
- ( ) no
- ( ) Other:

Would you like to become involved in the CONSORT EHEALTH group?

This would involve for example becoming involved in participating in a workshop and writing an "Explanation and Elaboration" document

- ( ) yes
- (x) no
- ( ) Other:

Clear selection

Any other comments or questions on CONSORT EHEALTH

Your answer

STOP - Save this form as PDF before you click submit

To generate a record that you filled in this form, we recommend to generate a PDF of this page (on a Mac, simply select "print" and then select "print as PDF") before you submit it.

When you submit your (revised) paper to JMIR, please upload the PDF as supplementary file.

Don't worry if some text in the textboxes is cut off, as we still have the complete information in our database. Thank you!

Final step: Click submit !

Click submit so we have your answers in our database!

Submit Clear form

Never submit passwords through Google Forms.

This content is neither created nor endorsed by Google. Report Abuse - Terms of Service - Privacy Policy

Google Forms
