# Supplementary material for: Impacts of GlobalConsent, a Web-Based Social Norms Edutainment Program, on Sexually Violent Behavior and Bystander Behavior Among University Men in Vietnam: Randomized Controlled Trial
Source: JMIR Public Health Surveill. 2023 Jan 27;9:e35116. doi: 10.2196/35116 (PMC9919511; doi:10.2196/35116)
Supplement: Multimedia Appendix 2 [file publichealth_v9i1e35116_app2.pdf]

Appendix 1. GlobalConsent and Youth Health Screenshots

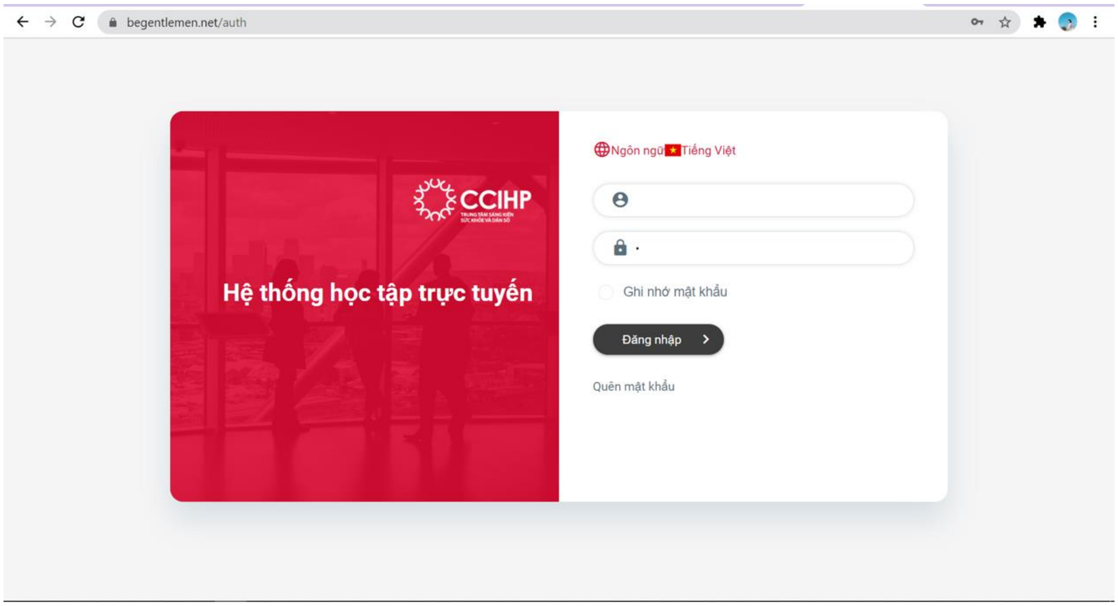

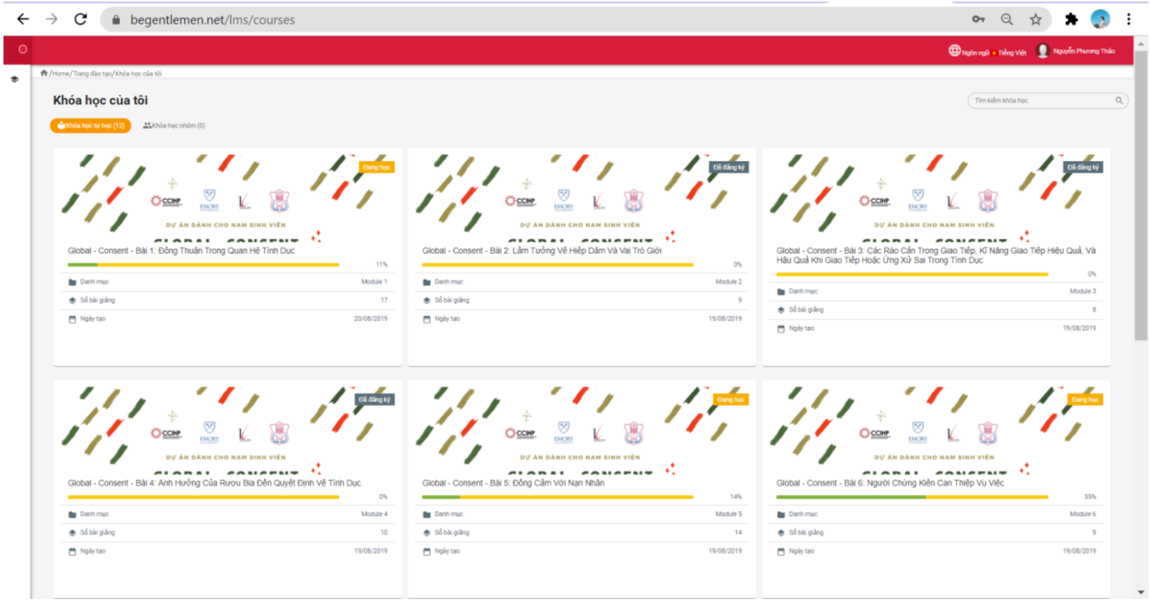

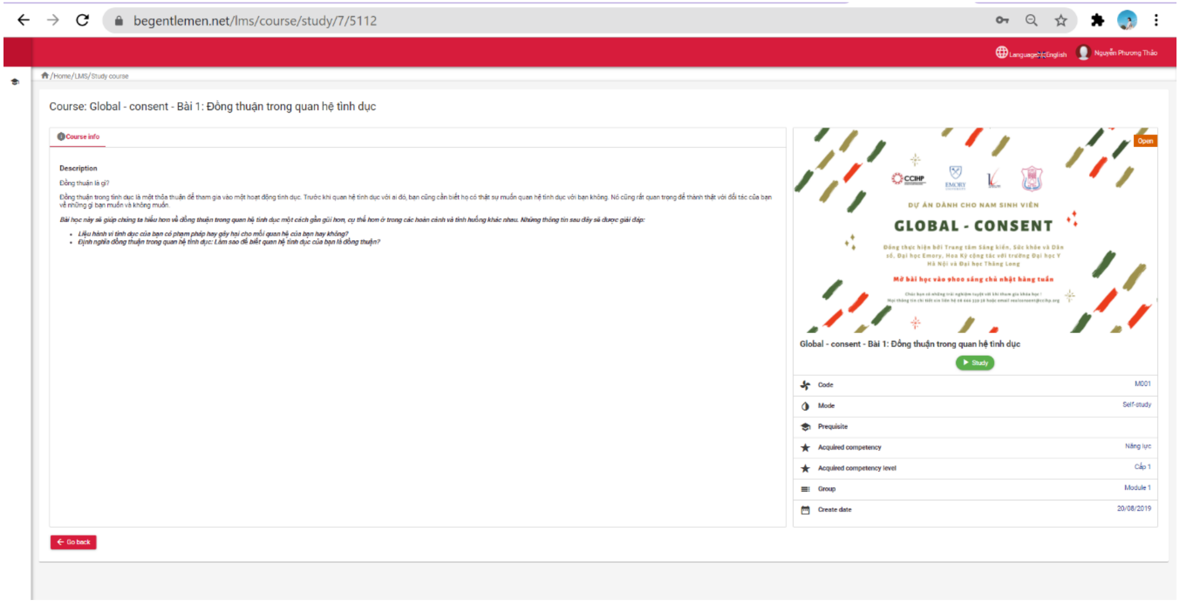

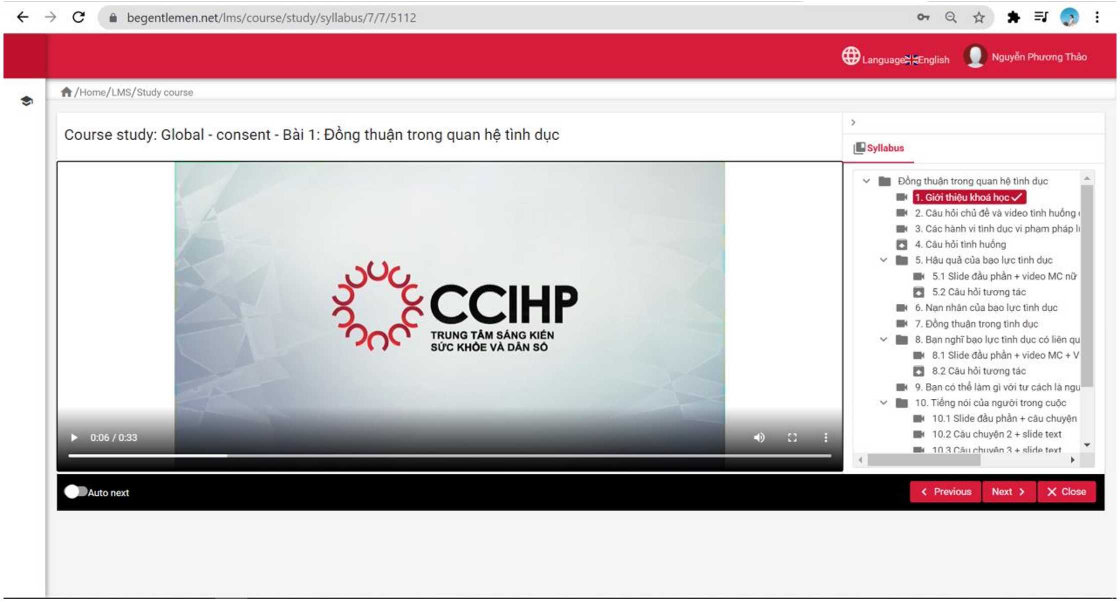

begentlemen.net/lms/course/study/syllabus/7/7/5112
Language English
Nguyễn Phương Thảo
/Home/LMS/Study course
Course study: Global - consent - Bài 1: Đồng thuận trong quan hệ tình dục
Syllabus
Đồng thuận trong quan hệ tình dục
1. Giới thiệu khoá học
2. Câu hỏi chủ đề và video tình huống
3. Các hành vi tình dục vi phạm pháp l
4. Câu hỏi tình huống
5. Hậu quả của bạo lực tình dục
5.1 Slide đầu phần + video MC nữ
5.2 Câu hỏi tương tác
6. Nạn nhân của bạo lực tình dục
7. Đồng thuận trong tình dục
8. Bạn nghĩ bạo lực tình dục có liên qu
8.1 Slide đầu phần + video MC + V
8.2 Câu hỏi tương tác
9. Bạn có thể làm gì với tư cách là ngu
10. Tiếng nói của người trong cuộc
10.1 Slide đầu phần + câu chuyện
10.2 Câu chuyện 2 + slide text
CCIHP
TRUNG TÂM SÁNG KIẾN SỨC KHỎE VÀ DÂN SỐ
0:06 / 0:33
Auto next
Previous
Next
Close

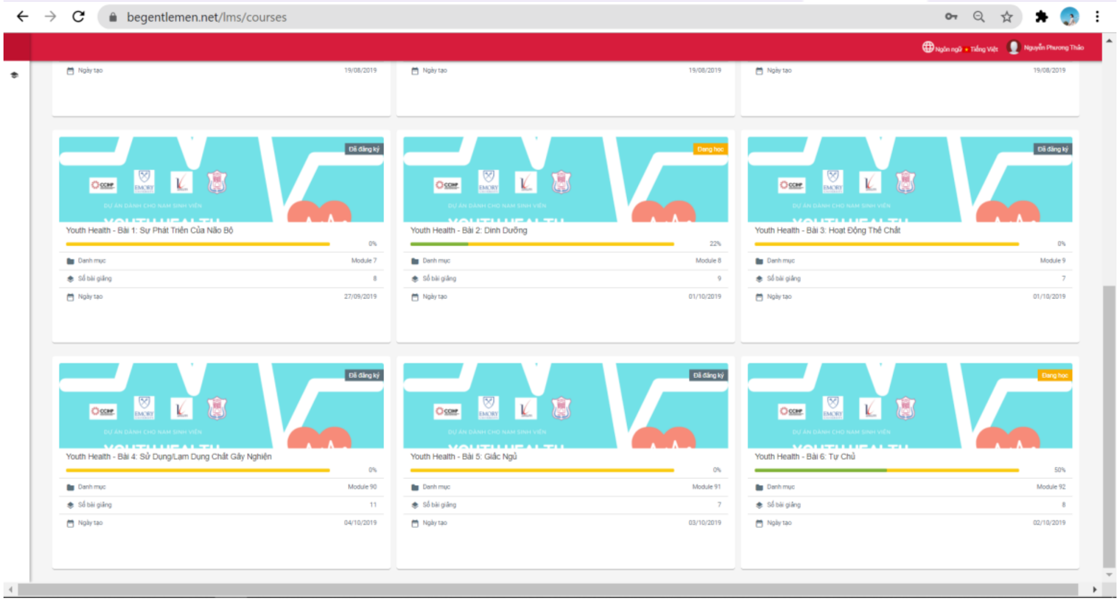

begentlemen.net/lms/courses
Youth Health - Bài 1: Sự Phát Triển Của Não Bộ
Youth Health - Bài 2: Dinh Dưỡng
Youth Health - Bài 3: Hoạt Động Thể Chất
Youth Health - Bài 4: Sử Dụng/Lạm Dụng Chất Gây Nghiện
Youth Health - Bài 5: Giấc Ngủ
Youth Health - Bài 6: Tự Chủ

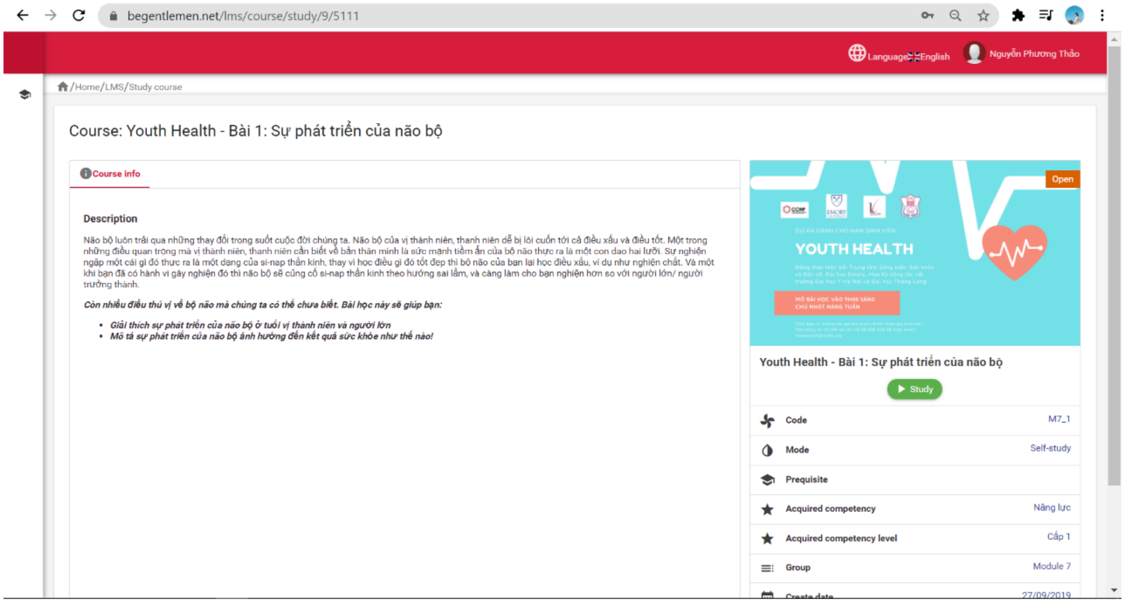

begentlemen.net/lms/course/study/9/5111
Language English
Nguyễn Phương Thảo
/Home/LMS/Study course
Course: Youth Health - Bài 1: Sự phát triển của não bộ
Course info
Description
Não bộ luôn trải qua những thay đổi trong suốt cuộc đời chúng ta. Não bộ của vị thành niên, thanh niên dễ bị lôi cuốn tới cả điều xấu và điều tốt. Một trong những điều quan trọng mà vị thành niên, thanh niên cần biết về bản thân mình là sức mạnh tiềm ẩn của bộ não thực ra là một con dao hai lưỡi. Sự nghiện ngập một cái gì đó thực ra là một dạng của si-nap thần kinh, thay vì học điều gì đó tốt đẹp thì bộ não của bạn lại học điều xấu, ví dụ như nghiện chất. Và một khi bạn đã có hành vi gây nghiện đó thì não bộ sẽ củng cố si-nap thần kinh theo hướng sai lầm, và càng làm cho bạn nghiện hơn so với người lớn/ người trưởng thành.
Còn nhiều điều thú vị về bộ não mà chúng ta có thể chưa biết. Bài học này sẽ giúp bạn:
Giải thích sự phát triển của não bộ ở tuổi vị thành niên và người lớn
Mô tả sự phát triển của não bộ ảnh hưởng đến kết quả sức khỏe như thế nào!
Open
YOUTH HEALTH
Youth Health - Bài 1: Sự phát triển của não bộ
Study
Code M7_1
Mode Self-study
Prequisite
Acquired competency Năng lực
Acquired competency level Cấp 1
Group Module 7
Create date 27/09/2019

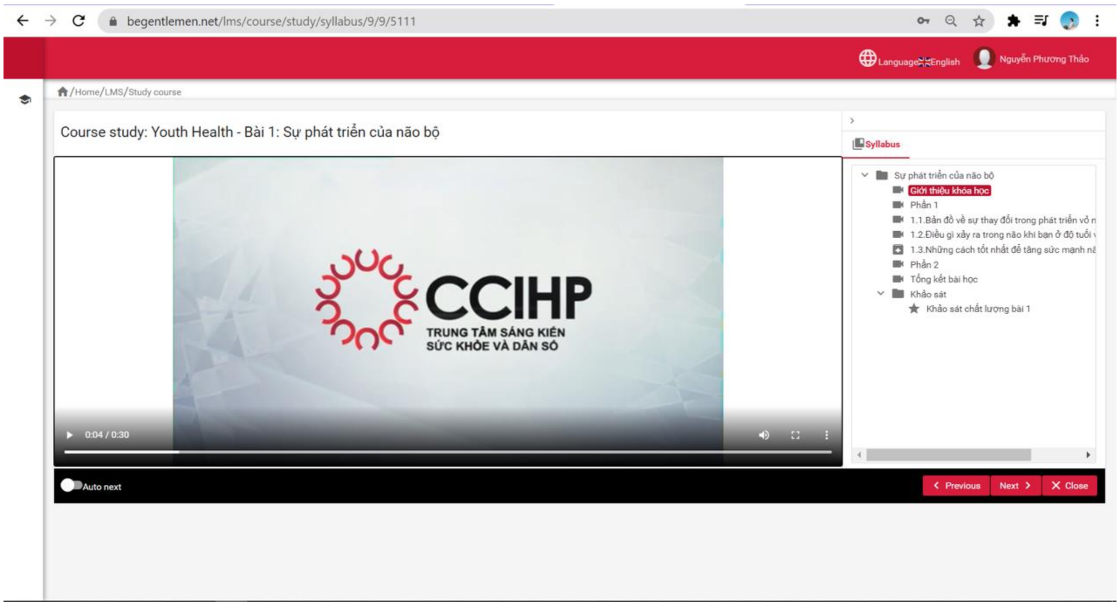
begentlemen.net/lms/course/study/syllabus/9/9/5111
Language English
Nguyễn Phương Thảo
/Home/LMS/Study course
Course study: Youth Health - Bài 1: Sự phát triển của não bộ
Syllabus
Sự phát triển của não bộ
Giới thiệu khóa học
Phần 1
Phần 2
Tổng kết bài học
Khảo sát
Khảo sát chất lượng bài 1
CCIHP
TRUNG TÂM SÁNG KIẾN SỨC KHỎE VÀ DÂN SỐ
0:04 / 0:30
Auto next
Previous
Next
Close

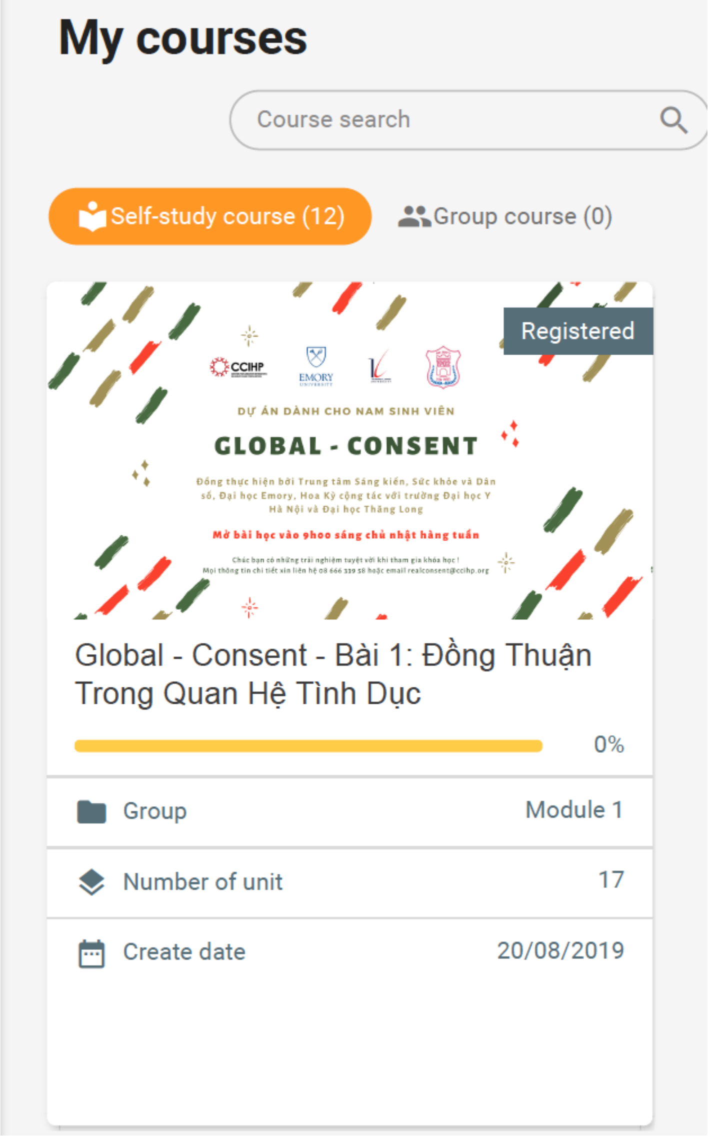
My courses
Course search
Self-study course (12)
Group course (0)
Registered
DỰ ÁN DÀNH CHO NAM SINH VIÊN
GLOBAL - CONSENT
Global - Consent - Bài 1: Đồng Thuận Trong Quan Hệ Tình Dục
0%
Group
Module 1
Number of unit
17
Create date
20/08/2019

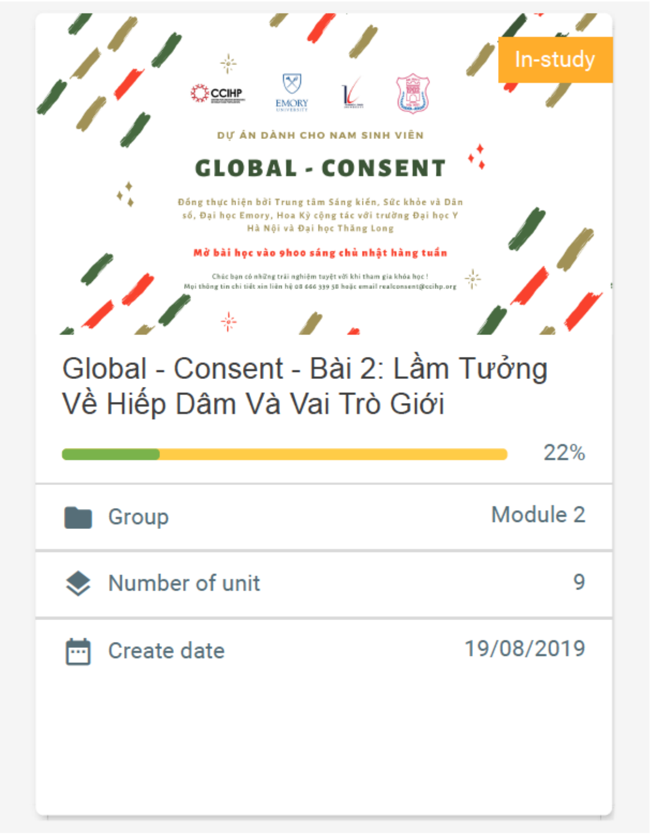
In-study
DỰ ÁN DÀNH CHO NAM SINH VIÊN
GLOBAL - CONSENT
Global - Consent - Bài 2: Lầm Tưởng Về Hiếp Dâm Và Vai Trò Giới
22%
Group
Module 2
Number of unit
9
Create date
19/08/2019

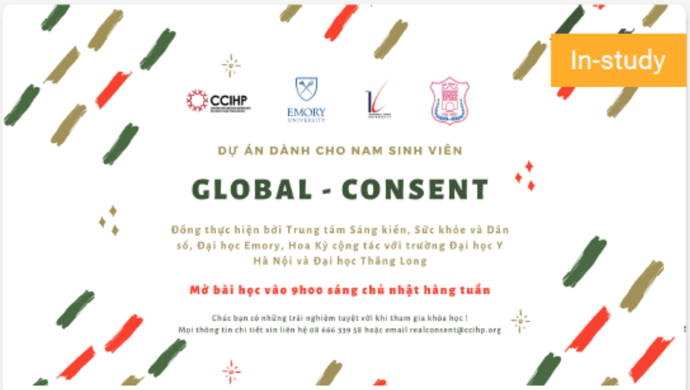

In-study

Global - Consent - Bài 3: Các Rào Cản Trong Giao Tiếp, Kĩ Năng Giao Tiếp Hiệu Quả, Và Hậu Quả Khi Giao Tiếp Hoặc Ứng Xử Sai Trong Tình Dục

0%

Group	Module 3
Number of unit	8
Create date	19/08/2019

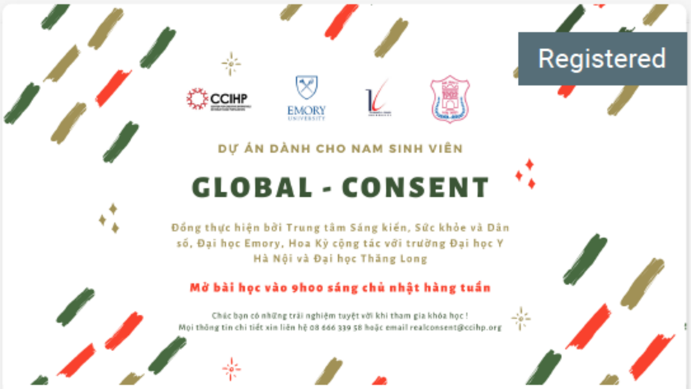

Registered

Global - Consent - Bài 4: Ảnh Hưởng Của Rượu Bia Đến Quyết Định Về Tình Dục

0%

Group	Module 4
Number of unit	10
Create date	19/08/2019

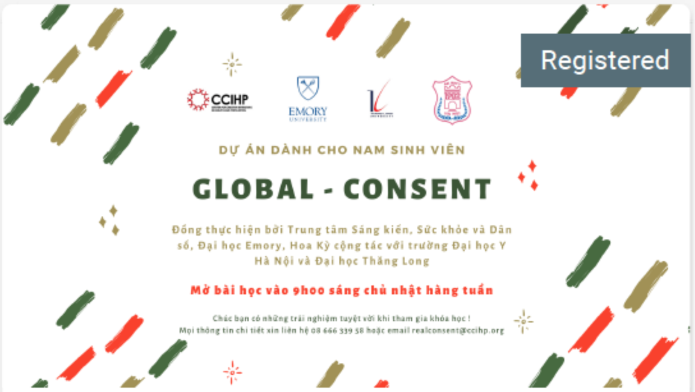

Registered

Global - Consent - Bài 5: Đồng Cảm Với Nạn Nhân

0%

Group	Module 5
Number of unit	14
Create date	19/08/2019

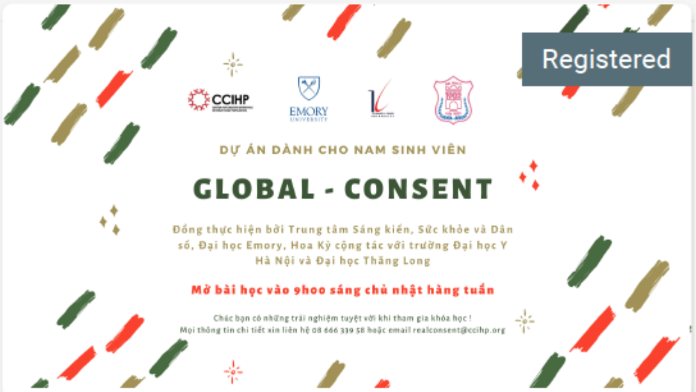

Registered

Global - Consent - Bài 6: Người Chứng Kiến Can Thiệp Vụ Việc

0%

Group	Module 6
Number of unit	9
Create date	19/08/2019

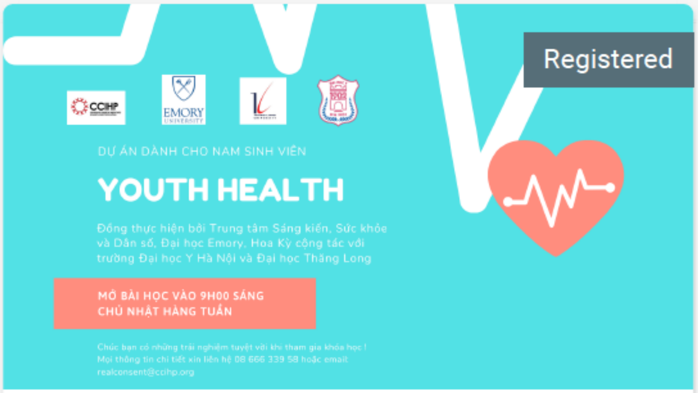

Youth Health - Bài 1: Sự Phát Triển Của Não Bộ

0%

Group	Module 7
Number of unit	8
Create date	27/09/2019

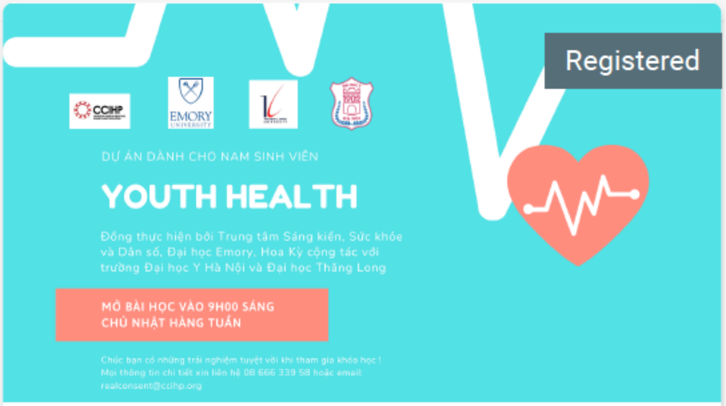

Youth Health - Bài 2: Dinh Dưỡng

0%

Group	Module 8
Number of unit	9
Create date	01/10/2019

Registered

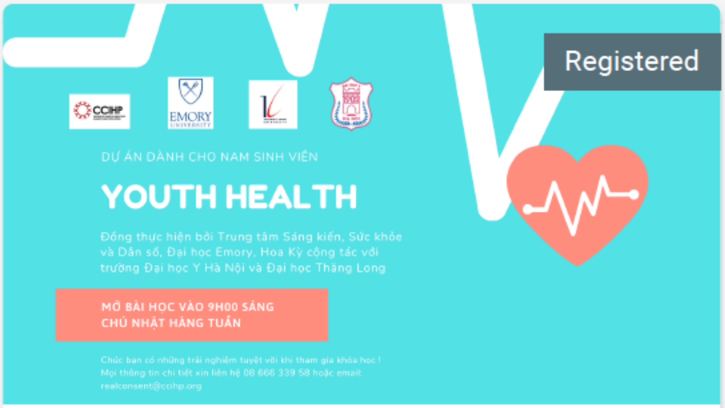

Youth Health - Bài 3: Hoạt Động Thể Chất

0%

Group	Module 9
Number of unit	7
Create date	01/10/2019

Registered

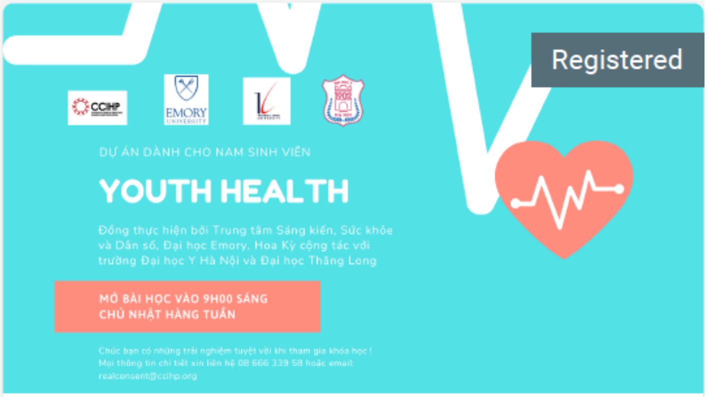

Youth Health - Bài 4: Sử Dụng/Lạm Dụng Chất Gây Nghiện

0%

Group	Module 90
Number of unit	11
Create date	04/10/2019

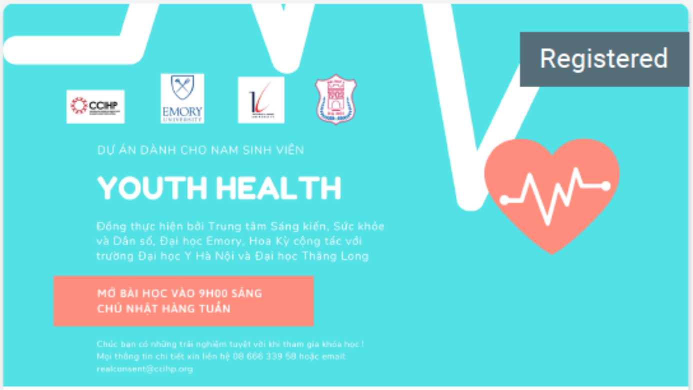

Youth Health - Bài 5: Giấc Ngủ

0%

Group Module 91

Number of unit 7

Create date 03/10/2019

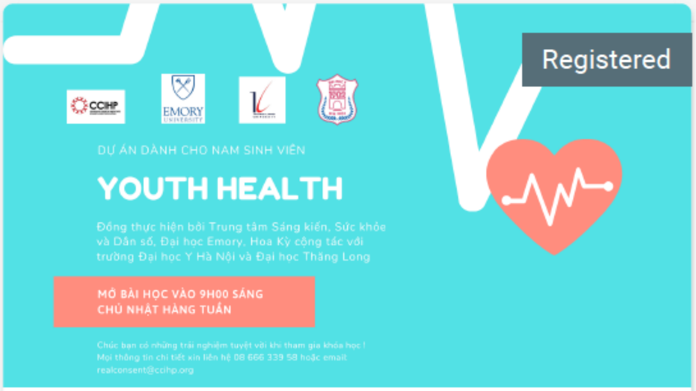

Youth Health - Bài 6: Tự Chủ

0%

Group Module 92

Number of unit 8

Create date 02/10/2019
