# Supplementary material for: Impacts of GlobalConsent, a Web-Based Social Norms Edutainment Program, on Sexually Violent Behavior and Bystander Behavior Among University Men in Vietnam: Randomized Controlled Trial
Source: JMIR Public Health Surveill. 2023 Jan 27;9:e35116. doi: 10.2196/35116 (PMC9919511; doi:10.2196/35116)
Supplement: Multimedia Appendix 3 [file publichealth_v9i1e35116_app3.docx]

**Multimedia Appendix 3: Documentation of Adaption of RealConsent to Create GlobalConsent**

The revision of the original Real Consent was made according to the qualitative findings and recommendations from male students and stakeholders on the original Real Consent and the adaptive storyboard for Global Consent.

The revision was made both for the structure of the Global Consent in general, and for the details in the transcript for each adaptive module in comparison with the original Real Consent

**General changes in terms of structure and presentation of the modules**

In terms of structure the key changes were in

1. **Development of main characters**. Character development of the four key male characters was adapted, according to qualitative findings from the semi-structural interviews. Specifically, GlobalConsent includes:
   1. 1 man with positive masculinity
   2. 1 with somewhat positive masculinity
   3. 1 with somewhat more traditional masculinity and becoming more aware in the middle of program (from module 4)
   4. 1 – more traditional man, struggling to become more aware at the end of program.

In addition, the female supportive characters were developed in pairs with the male characters, also based on qualitative findings from the semi-structured interviews that diversified narratives of sexual violence and gave nuances to the story development flow.

1. **Removal of coach-talk segments**, a behavioral-change technique of reinforced learning and identification with characters. These short segments, that relied heavily on colloquial English, were not well understood in Vietnamese culture and caused confusion for the audience regarding take-away messages of segments and scenarios.
2. **Addition of key questions and take-away messages**. Clear introductory messages about the purpose of segments or scenarios and clear take-away messages after segments or scenarios were added, as Vietnamese students preferred to have a clear message rather than to synthesize the information and narratives by themselves. This strategy also reduced variation in misinterpretation of key messages among program participants.
3. **Adaptation of information and data** as needed for Vietnam. Examples of adaptions included the content of legal documents/laws on sexual violence, alcohol and substance abuse; real cases of “rapists”; data of sexual violence cases.
4. **All scenarios were “re-filmed”** or turned to animation to be specific for Vietnam, and respond learning style of the male students (clear messages rather than metaphor use)

**Summary table of the adaptation**

| **Module** | **Topic** | **Description (Original RC)** | **Adaptation (GC)** | **Description of GC** |
| --- | --- | --- | --- | --- |
| **1** | **Consent for sex** | Serial drama episodes of young men discussing challenges of obtaining effective consent for sex; expert discussing the 4 elements of effective consent for sex; interactive segments presenting sexual scenarios related to the 4 elements of consent: young men are asked to indicate whether consent was obtained or possible, then feedback on their choices is provided; videos of real victims’ stories of being raped. | Key questions were added before the drama episodes and expert’s talk to help the audience more focused.  Narrative appeared in the first episode was replaced by newly happened case of sexual harassment in Vietnam showbiz. Context for the men’s conversation was adapted to local lifestyle e.g., men were gathering in teashop, karaoke service shop.  Interactive segments provide explanation for correct or incorrect choice/solution for men was added for a clearer message (action oriented).  Information on legal definitions of sexual violence and illegal sexual acts was modified according to Vietnam Law. Two new types of sexual violence related to cyber violence and sexual attack through SMS (sexual texting = sexting) with narrative taken from SSI with female students | Serial drama episodes of (1) young men discussing challenges of obtaining effective consent for sex, including a specific case of sexual harassment in Vietnam showbiz, and context of conversation adapted to Hanoi gathering lifestyle in teashops and karaoke service shops; (2) expert discussing the 4 elements of effective consent for sex where key questions and key words were added for a more focused take-home message; (3) interactive segments presented sexual scenarios related to the 4 elements of consent with additional explanation for correct or incorrect option that audience may have for a clearer and more action-oriented message; and (4) information on legal definition of sexual violence and illegal sexual acts according to Vietnam Law, cyber violence and sexual attack through SMS were added with narrative taken from qualitative interviews with female students. |
| **2** | **Rape myths, gender roles** | Serial drama episodes of young men discussing women who they know who were raped; interactive activity of male narrator discussing 6 main rape myths and debunking each of them; video of male narrator describing the socialization process for men—“ridiculous reality” and how it contributes to sexism and VAW; female narrator describing socialization process for women and how it contributes to a culture of sexism; interactive game where several profiles of young men are provided with images and men have to choose, “who is the rapist.” | Narrative appeared in the first drama episode was modified following a real case which emerged from individual interviews with female students (from HMU). Key question was added before the episode. Context of conversation was modified to match lifestyle: e.g., men were watching soccer match and talking  Interactive activity was modified in the way to discuss rape myths: instead of statement on men’s or women’s views on sexual violence, general concept of sexual violence highlighted the interaction between how men/women perceive and react to violence and the wider context of social expectations on their (gender) roles. Correct option with explanation was provided at the end of each question, responding the feedbacks from male students during storyboard development and testing that they need a clear message and correct knowledge.  The video of male and female narrators described the socialization process for men and women: added some sentences on the conflicting messages on the expected gender roles in a transitioning society, highlight that both men and women are negatively influenced by traditional masculinity (they are kind-of both “victims” and “actors” at the same time).  Profile of the rapists was replaced by the real cases in Vietnam (the ones were investigated and sent to the court). | Serial drama episodes of (1) young men discussing women who they know who were raped, that reflected a real case emerged from qualitative research with female students, and men’s idol of soccer player (gathering for soccer match watching); (2) interactive activity of male narrator discussing 6 main rape myths and debunking each of them, where highlight was the interaction between how men/women perceive and react to violence and the wider context of social expectations on their (gender) roles, and correct option with explanation was added at the end for clear take-home messages; (3) video of male narrator describing the socialization process for men—“ridiculous reality” and how it contributes to sexism and VAW: the conflicting messages on the expected gender roles in a transitioning society were emphasized to highlight negative influences of traditional masculinity to both men and women; and (4) interactive game “who is the rapist” where several profiles of the investigated cases of rape in Vietnam, including high reputation men as rapists and men and children as victims, are provided with images and men have to choose. |
| **3** | **Effective communication** | Serial drama episodes of young men discussing challenges of communication with a young woman to have sex and how to approach; segment describing young man and woman communicating all night to show inaccurate cue perceptions, then showing correction through communication; videos of young women describing what they want in terms of communication; interactive segment w/sexual scenarios and questions around consent, alcohol, and communication. | Little modification was made for module 3.  In the video of young women talking what they wanted in terms of communication (Segment 3.5) editing was made to address some of the norms e.g., girls don’t want a guy to be too pushy and that no means no, or that it is important to communicate about what girls want sexually. Besides, qualitative interviews (with female students) and group discussion suggested adaptation of the context for girls’ conversation as girls in Vietnam may not talk directly on sexuality, and rather more comfortable on SMS. Instead of face-to-face discussion, the girls have group texting. While a couple girls met in person at a café, one gets a text and then they all talk (via messenger).  Consent in a flirting period was added to the portfolio, e.g., bomb of flirting SMS (segment 3.5), 2^nd^ kiss and fooling around in flirting period (much thinner line between consent vs. unconsent) (segment 3.6)  Interactive segment with sexual scenarios and questions around consent, alcohol and communication (segment 3.6): Addressing findings from qualitative interviews with female students, (1) we added consent for the second (or N) time of sex between lovers who already had sex many times; (2) we highlighted girl’s asking about safe sex with the guy in the past in case of both long-term loving relationship and unidentified relationship. | Serial drama episodes of (1) young men discussing challenges of communication with a young woman to have sex and how to approach where some of norms were addressed e.g., girls don’t want a guy to be too pushy and that no means no, or that it is important to communicate about what girls want sexually; (2) segment describing young man and woman communicating all night to show inaccurate cue perceptions, then showing correction through communication; (3) videos of young women describing what they want in terms of communication that adapted with format of group texting that was reported more comfortable for girls in Vietnam given culture considering sex as sensitive topic for face-to-face talk; (4) interactive segment w/sexual scenarios and questions around consent, alcohol, and communication where consent in a flirting period and not-first-time sex between lovers were added to highlight the thin line between consensual and coercive sex in long-term (or committed) relationships. |
| **4** | **Alcohol and rape** | Serial drama episodes of young men at a house party with alcohol being served and modeling responsible behavior (e.g., deciding not to have sex); interactive segment that is a quiz with feedback given covering the physical, emotional and cognitive effects of alcohol; interactive segment of sexual scenarios involving alcohol and whether consent is possible; segment providing normative feedback on binge drinking among college students; 1st person video of young man going out for the evening and drinking alcohol and the negative outcomes. | The adaptation for module 4 mostly about regulation of alcohol use, rape law and sentencing in Vietnam.  Besides, minor adaptation was made for segment 4.7 on the context of a birthday party for all scenarios and the last video clip filmed in context of a street café instead of apartment. Key words appeared on slides for the interactive segment for audience better capture. | Serial drama episodes of (1) young men at a birthday party with alcohol being served and modeling responsible behavior (e.g., deciding not to have sex); (2) interactive segment that is a quiz with feedback given covering the physical, emotional and cognitive effects of alcohol, and regulation of alcohol use in Vietnam; (3) interactive segment of sexual scenarios involving alcohol and whether consent is possible, with added information on rape law and sentencing in Vietnam; (4) segment providing normative feedback on binge drinking among college students; and (5) 1st person video of young man going out for the evening and drinking alcohol and the negative outcomes. |
| **5** | **Victim empathy** | Serial drama episodes of young men discussing women’s rape experiences and men’s rape experiences in context of a rape that occurred on campus; segment highlighting inaccurate statements about rape and providing true information; video of expert discussing nuances of coercion and coercive behavior with 3 young men; real stories from a young man and young woman describing their sexual assault/rape; video clips of people providing tips on how to help a survivor. | Similar to all other modules, introduction or key questions were added before episodes and interactive segment to help audience more focused; and key points at the end for take-home message.  Segment 5.3 changed the format to form of a short quiz instead of table for better interaction. We added 1 question on the case when victim did not fight back to challenge the popular myth of rape in Vietnam.  Segment 5.6 we made signification revision with detail of authorities’ response to report of sexual violence in story 1 (to address the situation of poor belief in supportive system in Vietnam), and adapted stories 3 and 4 on coercive sex in a loving relationship, and sexual text bombing in a flirting period. | Serial drama episodes of (1) young men discussing women’s rape experiences and men’s rape experiences in context of a rape that occurred in students’ relationships; (2) segment highlighting inaccurate statements about rape and providing true information in an adapted format of short quiz, with one additional case when victim did not fight back to challenge the popular myth of rape in Vietnam; (3) video of expert discussing nuances of coercion and coercive behavior with 3 young men; (4) real stories from a young man and 3 young women describing their sexual assault/rape, where were addressed authorities’ poor response to report of sexual violence, coercive sex in a loving relationship, and sexual text bombing in a flirting period; (5) video clips of people providing tips on how to help a survivor. |
| **6** | **Bystander intervention** | Serial drama episodes of young men discussing the idea of pluralistic ignorance and doing something rather than nothing to stop a guy from being violent; video of expert discussing the barriers to intervening, safe and effective ways to intervene, and benefits to intervening; videos of 3 people who were in a situation where they did not intervene with discussion on the negative consequences; 3 videos with narration by male narrator of men behaving badly toward women and then illustrating how to intervene with positive outcomes shown. | Similar to all other modules, introduction or key questions were added before episodes and interactive segment to help audience more focused.  In the third video with narrator, a small modification was made for the context of bad behavior that we used story of a female student for illustration of sexual harassment and poor protection mechanism of the working place (during internship).  In the last segment we added supportive resources for victims of sexual violence in Hanoi (both online and face-to-face support) | Serial drama episodes of (1) young men discussing the idea of pluralistic ignorance and doing something rather than nothing to stop a guy from being violent; (2) video of expert discussing the barriers to intervening, safe and effective ways to intervene, and benefits to intervening; (3) videos of 3 people who were in a situation where they did not intervene with discussion on the negative consequences, where story of a female student was used to illustrate case of sexual harassment in the working place (during internship); (4) 3 videos with narration by male narrator of men behaving badly toward women and then illustrating how to intervene with positive outcomes shown. And the last segment provided supportive resources for both victims and men in general (bystanders) of sexual violence in Hanoi (both online and face-to-face support). |

**Module 1:**

Module 1 involved the biggest changes in comparison to the rest of the program. Reasons: (1) this module provided the audience with legal definitions of sexual violence, illegal sexual acts; (2) data on sexual violence and rape. In addition, (3) we updated the program by adding two new types of sexual violence related to cyber violence and sexual attack through SMS (sexual texting = sexting).

Objectives: We kept the main meanings of the objectives; however, we changed the order to match the flow of the content of the module, and the statement way to address the view of audience (talk from audience perspective). E.g., instead of saying “Understand how sexual assault of women affect all men”, the objective was rephrased as “being sure if your sexual behaviors are illegal or harmful”.

First scenario:

- We added key question before the first scenario
- We changed the scene of TV watching into gathering in a teashop and look through Facebook that more common/popular scene to male students in Vietnam today. The newly happened case of sexual harassment in Vietnam showbiz was replaced the original introduction story.

Segment on illegal and harmful sexual acts

- All the information on legal regulation/law was replaced by the one in Vietnam. The distinction between illegal and harmful sexual acts were kept the same
- We added 5 questions on having non-consensual sex without the use of physical force, on filming and dissemination of sex clip (of their own), on harmful but not yet illegal sexual behaviors, on non-consensual sexual texting, on rape without physical injuries. Those questions were decided according to the narratives found from SSI with male and female students
- We took out the question on policy on rape in university as qualitative data showed that both universities have no such policy

Segment on data of sexual violence

- Changed to data available in Vietnam

Segment on Alan talk (talk by expert)

- Added key questions and introduction of 4 elements of informed consent (in slide) to be more visible to the audience
- Key content kept the same

Segment on how sexual violence related to audience (male students)

- Key question was added
- Scenario context was changed from university campus to karaoke service shop as it is more popular/ familiar to male students in Vietnam (found from SSI and FGD)

Segment on scenarios analysis

- Key question was added
- Scenario context was changed to be more cultural and local context specific: from party of students’ association to karaoke service shop and birthday party, from going by car to going by motorbike
- In the explanation for correct or incorrect choice (of the interactive questions at the end of scenarios), correct solution for the man was added for a clearer message (action oriented)

Segment on acts of bystanders:

- Key question was added
- Format was changed from puppet conversation (metaphor was hard to understand) to scene with real persons
- Key message: visible as a slide and correct solution for the man was added for a clearer message (action oriented)

Segment on voice of insiders (victims of sexual violence)

- Key question was added
- Key messages on consequences to the victims were more visible to audience – appearing as a slide after each story
- Story 2 was modified with details from SSI with female students
- Story 4 was replaced by a new one on reaction to boyfriend’s non-consensual first kiss. The original RC mentioned girl’s frightening due to “big body” of the man, which is not appropriate to Asian male students’ physical characteristics.
- A new story on sexual texting bomb by a crush was added to be more consistent to the segment on harmful (but not yet illegal) sexual acts, and it also emerged from SSI with female students

Last scenario

- We exchanged story by Ellen in the original RC to another girl, and exchanged some conversation lines between Eric and Alex that help story matched with new characteristics development (as mentioned above)
- Key question was added

**Module 2:**

The biggest changes in module 2 are (1) the replacement of real cases of rape which either emerged from individual interviews with female students or from mass media (report from the court); (2) change the way to present sexual violence myths from talking about some men/women words or reactions, to a wider context of social expectations that they are always having interaction with.

Objectives:

- We added some words to make meanings clearer. E.g., instead of saying “illustrate myths about rape”, we said “illustrate myths and realities about…”
- We changed the way we presented the last objective talking about the connection between social expectations of gender performance and sexual violence, instead of the connection between men’s words and their actions. Reason: (1) better flow from objective 2 on social expectations to objective 3; (2) wider view on the context (social expectations) of men’s words and actions.

Segment 2.2. – Introduction (first clip)

- We added key questions before clip to help audience focusing on myths of sexual violence
- We changed the narrative on the clip to a real case which was emerged from individual interviews with female students (from HMU)

Segment 2.4.

- We kept the format of myths’ challenge with a statement/question and interactive choices for audience below. However, as mentioned above, we changed the way to present the myths from perspectives of some men/women to concept of sexual violence in general, that highlight the interaction between how men/women perceive and react to violence and the wider context of social expectations on their (gender) roles. This also helped to avoid any possible biased interpretation due to audience’s assumption on the men/women mentioned in the question.
- Besides, we also provided “correct answer” with explanation at the end of each interactive question/statement. This change responded the feedbacks from male students during storyboard development and testing that they need a clear message and correct knowledge

Segment 2.5.: changed to real rapists (reported and brought out to the court) in Vietnam

Segment 2.6.:

- Key questions added before clip
- Added some sentences on the conflicting messages on the expected gender roles in a transitioning society, highlight that both men and women are negatively influenced by traditional masculinity (they are kind-of both “victims” and “actors” at the same time)

Segment 2.8.

- Changed the context of conversation between the guys to a more popular scenario of male students in Vietnam (watching and talking about soccer match)
