# Supplementary figures and images for: Impacts of GlobalConsent, a Web-Based Social Norms Edutainment Program, on Sexually Violent Behavior and Bystander Behavior Among University Men in Vietnam: Randomized Controlled Trial
Source: JMIR Public Health Surveill. 2023 Jan 27;9:e35116. doi: 10.2196/35116 (PMC9919511; doi:10.2196/35116)

**Multimedia Appendix 4: Kernel Density Plots of Count Outcomes**

**
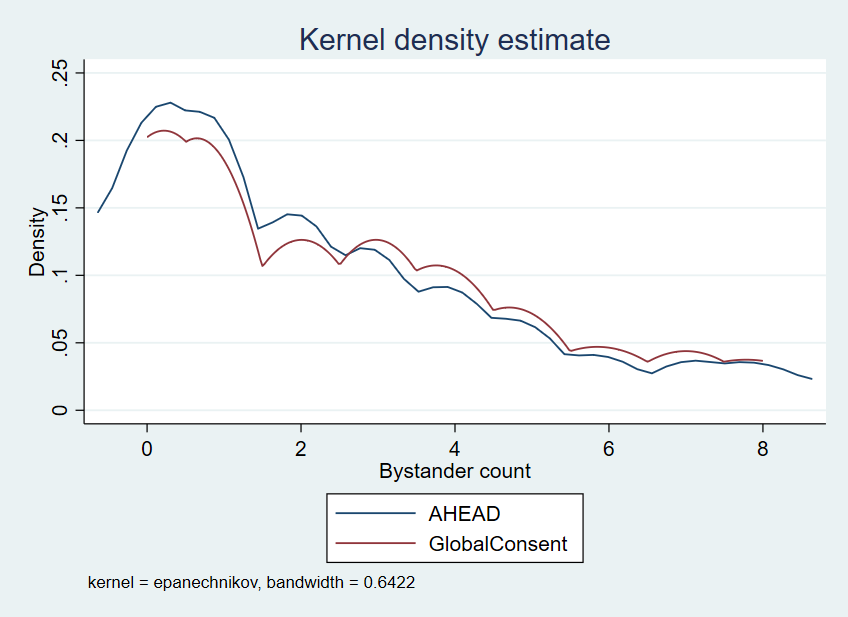
**

**
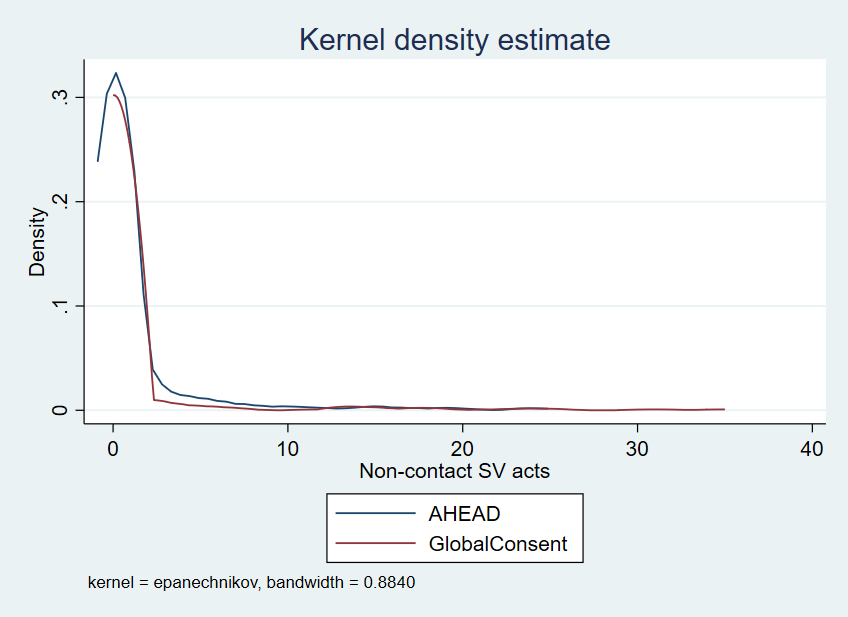
**

**
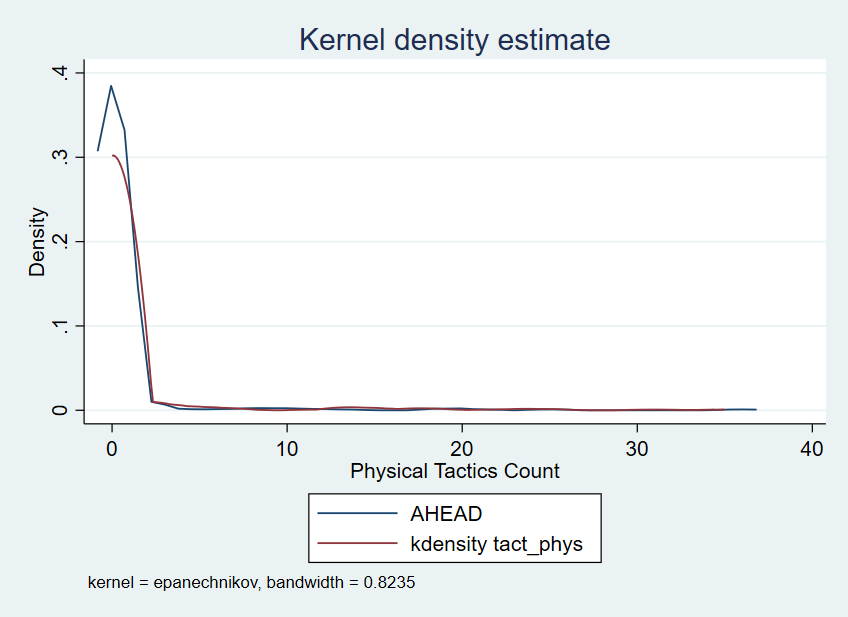
**

**
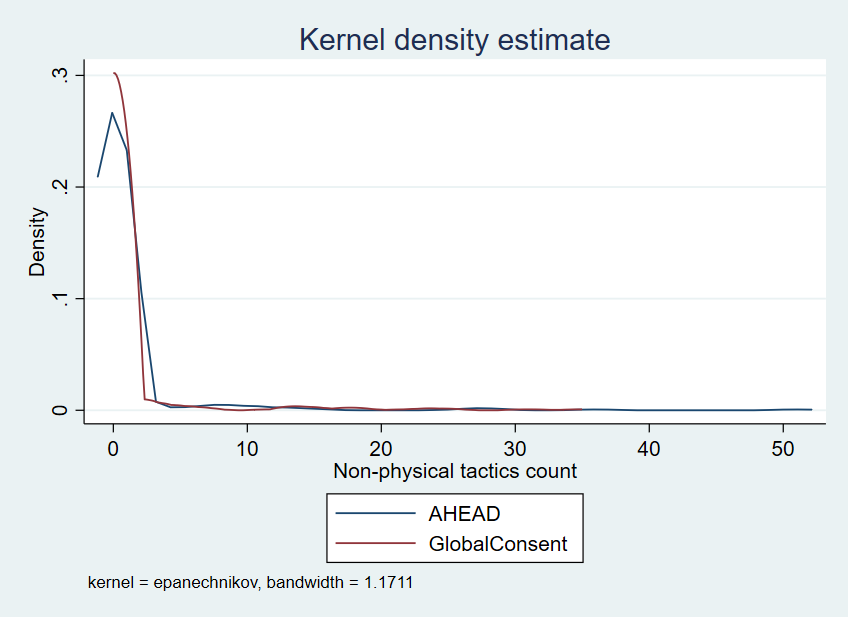
**

Supplement: Multimedia Appendix 4 [file publichealth_v9i1e35116_app4.docx]
